# Diet-based weight-loss intervention is not associated with a meaningful change in lean soft tissue

**DOI:** 10.1016/j.ajcnut.2026.101251

**Published:** 2026-02-23

**Authors:** Aubrey K Roberts, Daniel J Panyard, Brady Hislop, Catherine P Ward, Michael P Snyder, Christopher D Gardner, Francois Haddad

**Affiliations:** 1 Department of Epidemiology and Population Health, Stanford University School of Medicine, Stanford, CA, United States; 2 Department of Genetics, Stanford University School of Medicine, Stanford, CA, United States; 3 Stanford Prevention Research Center, Department of Medicine, Stanford University School of Medicine, Stanford, CA, United States; 4 Division of Cardiovascular Medicine, Stanford University School of Medicine, Stanford, CA, United States

**Keywords:** body composition, weight loss, lean soft tissue, lean mass, muscle mass, diet intervention, proteomics

## Abstract

**Background:**

An emerging concern is that weight-loss interventions can lead to disproportionate muscle loss. Few studies accurately quantify changes in lean soft tissue (LST) after weight loss or investigate associated molecular signatures.

**Objectives:**

The objectives of this study were to quantify LST change after a diet-based weight-loss intervention and identify protein biomarkers associated with LST retention.

**Methods:**

Using the Diet Intervention Examining The Factors Interacting with Treatment Success cohort, we analyzed LST from dual-energy X-ray absorptiometry in three ways: *1*) by body region (appendicular and total body), *2*) after removing bias from fat-free adipose tissue (FFAT), and *3*) relative to body size (percentage predicted LST). We also assessed 242 proteins measured in Olink Cardiovascular II, III, and Inflammation panels as predictors of LST change.

**Results:**

A total of 374 participants (61% female; mean age ± standard deviation (SD): 39.4 ± 6.7 y; mean body mass index ± SD: 32.3 ± 3.2 kg/m^2^) who had been randomly assigned to healthy low-fat or low-carbohydrate diets were pooled and analyzed at baseline and 6 mo. Total mass changed by −5.9 kg (95% confidence interval [CI]: −6.51, −5.29) in females and −7.18 kg (95% CI: −8.2, −6.16) in males. Appendicular LST change was modest at −0.80 kg (95% CI: −0.92, −0.69) in females and −1.02 kg (95% CI: −1.22, −0.83) in males. Appendicular LST losses comprised <10% of total mass loss after adjusting for FFAT. Appendicular LST relative to body size also increased at 6 mo (*P* < 0.001). Changes in 10 proteins in females and 27 in males predicted LST change (5% false discovery rate), with protein delta homolog 1 (DLK1)—an inhibitor of adipogenesis—as the top predictor.

**Conclusions:**

Change in appendicular LST, a surrogate for skeletal muscle, was modest after 6 mo of diet-based weight loss. DLK1, an inhibitor of adipogenesis, emerged as the top protein biomarker linked to LST retention.

This trial was registered at clinicaltrials.gov as NCT01826591.

## Introduction

Weight-loss interventions can lead to major improvements in body weight, insulin sensitivity, lipid profiles, and other health parameters [[1], [2], [3], [4], [5], [6]]. Despite these benefits, there is concern that rapid weight loss can lead to disproportionate muscle loss [7,8]. Diet-based weight-loss interventions show variable reductions in lean soft tissue (LST), ranging from 6% to ∼30% of total weight loss [9]. However, the magnitude of LST loss following diet-based interventions and its potential health consequences remain contested [7,8].

Two major limitations of existing clinical studies are that they use imperfect methods to measure “muscle mass” and rarely evaluate the clinical significance of observed changes. Although MRI is the gold standard for quantifying skeletal muscle, dual-energy X-ray absorptiometry (DXA) is more widely used because it is accessible and cost effective. However, DXA does not directly measure skeletal muscle; it measures 3 compartments: LST, fat mass (FM), and bone mineral content [10]. LST encompasses more than skeletal muscle, including internal organs, connective tissue, and fat-free adipose tissue (FFAT) [11]. Additionally, the composition of weight loss—LST vs. FM—can vary substantially across individuals, yet clinical studies rarely examine this heterogeneity or its underlying mechanisms. Existing studies have explored variability in weight loss and proteomic signatures of weight change, but have not distinguished proteins linked to LST changes from those related to FM [[12], [13], [14], [15]].

To address these methodological limitations, we assess changes in appendicular (arms and legs) LST, which is ∼70% skeletal muscle [16]. We also adjust for FFAT, a constituent of adipose tissue, in LST measurements [11]. Furthermore, we examine whether changes in LST are “proportional” to body size, providing insight into the clinical significance of LST changes. We also isolate protein biomarkers related to LST retention during weight loss.

**FIGURE 1:**
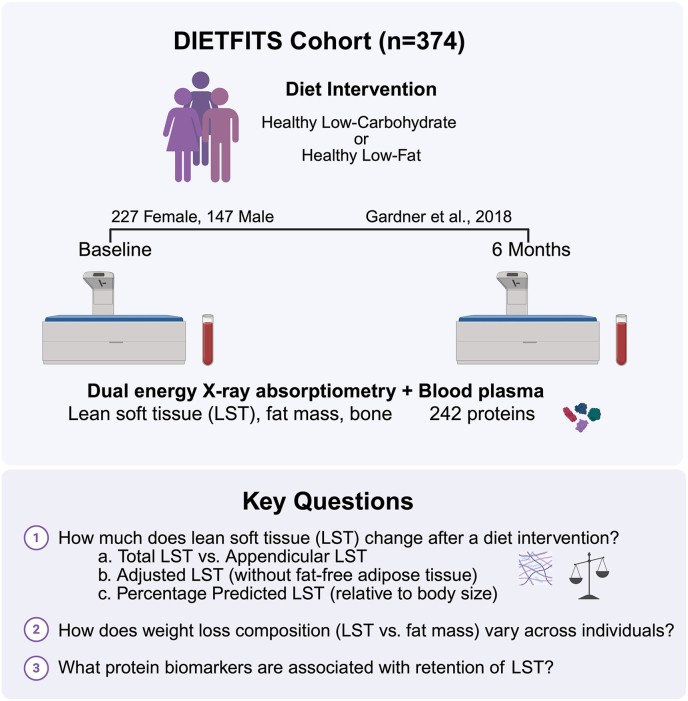
Overview of the study design and objectives. Created with BioRender.com. DIETFITS, Diet Intervention Examining the Factors Interacting with Treatment Success; LST, lean soft tissue.

In the present study, we explore changes in LST among 374 participants from the Diet Intervention Examining the Factors Interacting with Treatment Success (DIETFITS) study [1]. Our objectives were to: *1*) quantify changes in LST after 6 mo of diet-based weight loss by i) body region, ii) after adjusting for FFAT, and iii) relative to body size; *2*) examine how weight-loss composition (LST vs. FM) varies among individuals; and *3*) identify protein biomarkers associated with LST retention during weight loss (Figure 1).

## Methods

### DIETFITS dataset

We performed a secondary analysis of the DIETFITS dataset (NCT01826591) [1]. DIETFITS enrolled 609 adults with overweight or obesity, aged 18–50 y, without diabetes, and with a BMI between 28 and 40 kg/m^2^. Participants were randomly assigned to a 12-mo diet intervention: healthy low-fat (HLF) or healthy low-carbohydrate (HLC) [17]. DXA scans were completed using a Hologic QDR-4500W fan-beam scanner at baseline, 6 mo, and 12 mo. Blood plasma for Olink proteomic assays was also collected at baseline and 6 mo. Basic demographic information (sex, age, and race/ethnicity) was obtained at baseline. Total energy expenditure (TEE) (kcal/kg/d) was quantified using the Stanford Seven-Day Physical Activity Recall questionnaire at baseline, 3 mo, and 6 mo [18].

**FIGURE 2:**
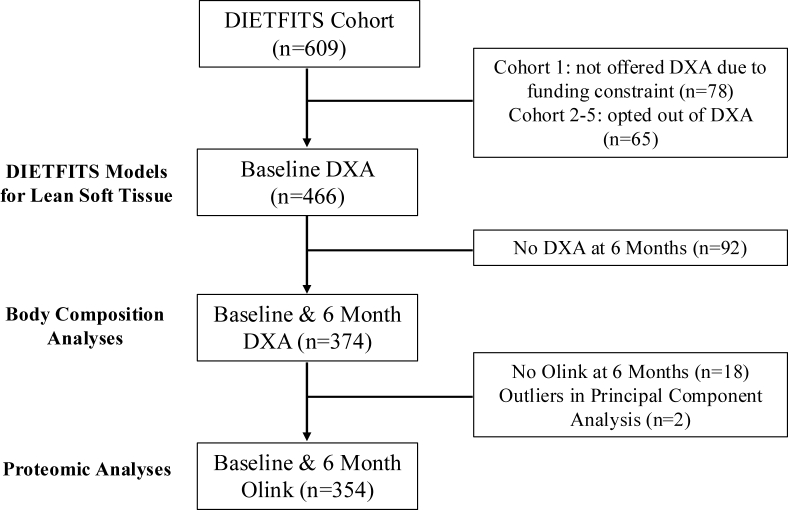
Participant flow diagram. The Diet Intervention Examining The Factors Interacting with Treatment Success (DIETFITS) cohort comprised 609 individuals who participated in a diet-based weight-loss intervention. Due to funding constraints, dual-energy X-ray absorptiometry (DXA) scans were offered only to cohorts 2 to 5. In total, 466 participants had DXA scans at baseline, and 374 had baseline and 6-mo scans. The majority of participants with DXA scans also completed baseline and 6-mo blood draws for proteomic assays (Olink) (*n* = 354).

Given that participants experienced the greatest weight loss in the first 6 mo, subsequent analyses focused on comparisons of DXA measures at baseline and 6 mo [1]. Due to funding constraints, DXA was not available for the first cohort of study participants [17] but was offered as an optional measurement for cohorts 2–5, resulting in 466 participants with baseline scans (Figure 2). A total of 374 participants had DXA data available at baseline and 6 mo (Figure 2). Of these, all had Olink proteomic data at baseline; only 18 participants did not return for follow-up blood collection and Olink assays at 6 mo (Figure 2).

### Adjusted DXA measurements: correcting for FFAT

**FIGURE 3:**
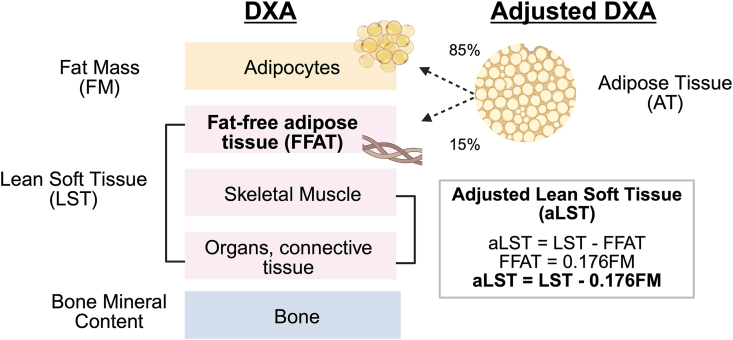
Dual-energy X-ray absorptiometry (DXA) compared with adjusted DXA measurements. DXA measures 3 components: fat mass (FM), lean soft tissue (LST), and bone mineral content. Fat-free adipose tissue (FFAT) comprises water, proteins, and minerals and is part of adipose tissue (AT), yet DXA classifies FFAT as LST. Adjusted LST (aLST) is a simple adjustment (aLST = LST − 0.176 FM) that may more closely approximate LST mass. Created with BioRender.com.

DXA measures 3 compartments: FM, LST, and bone mineral content (Figure 3). However, FM and LST (molecular compartments) from DXA are not equivalent to adipose tissue or skeletal muscle (tissue-organ compartments) [10]. DXA classifies FFAT as LST, even though FFAT is structurally part of adipose tissue. Adipose tissue is composed of ∼85% FM (adipocytes) and 15% FFAT (water, protein, and minerals) [19]. Using this information, we can estimate adipose tissue and FFAT amounts from DXA-derived FM and then calculate adjusted LST (aLST) [11]. aLST may more closely approximate LST mass without bias from FFAT (Figure 3, Supplemental Table 1).

### Percentage predicted LST

“Percentage predicted” metrics are used in clinical practice to compare an individual’s health with predicted values (e.g., percentage of age-predicted peak oxgyen uptake [ppVO_2_]) [20]. We applied this concept to LST to express it relative to body size and expected norms. We defined percentage predicted LST as follows:

(1) Percentage predicted LST = (observed LST / predicted LST) ∗ 100

Predicted LST was derived from NHANES anthropometric models by Lee et al. [21] (*R*^*2*^ = 0.85 for females and *R*^*2*^ = 0.88 for males), where height is expressed in cm and weight in kg:

(2) LST_Female_ = –14.292 – 0.046(Age) + 0.201(Height) + 0.347(Weight) – 0.448(I_Mexican_) – 0.047(I_Hispanic_) + 1.128(I_Black_) – 0.384(I_OtherRace_)

(3) LST_Male_ = –14.729 – 0.071(Age) + 0.210(Height) + 0.468(Weight) – 0.441(I_Mexican_) + 0.320(I_Hispanic_) + 1.821(I_Black_) – 0.784(I_OtherRace_)

### DIETFITS-derived models for appendicular LST

NHANES did not have a model for appendicular LST [21], so we derived models from the DIETFITS dataset. We then used predicted appendicular LST from DIETFITS-derived models to calculate percentage predicted appendicular LST as follows:

(4) Percentage predicted appendicular LST = (observed appendicular LST / predicted appendicular LST) ∗ 100

This approach allowed us to compare an individual’s appendicular LST with expected norms based on their height, weight, and demographics.

We used Least Absolute Shrinkage and Selection Operator (LASSO) regression (*glmnet* package in R) [22] to develop an allometric model for appendicular LST using baseline DXA data from DIETFITS (*n* = 466). Individuals with baseline DXA data (*n* = 466) had demographics similar to those with both baseline and 6-mo DXA data (*n* = 374) (Supplemental Table 2). Separate models were created for females (*n* = 276) and males (*n* = 190) using 80/20 train-test splits. Predictors included appendicular mass, height, age, age^2^, and binary variables for race (Black, Asian, or Other) and ethnicity (Hispanic), to match NHANES [21]. Appendicular LST, appendicular mass, and height were log-transformed. 10-fold cross-validation (*cv.glmnet*) was used to select the λ that minimized the training mean squared error [22]. A final LASSO model was fit to the training set with this λ. Model accuracy was assessed on the test set using *R*^*2*^ (Supplemental Figure 1). The same procedure was used to generate models for total LST to compare with NHANES LST models, using total mass instead of appendicular mass as a predictor.

### Proteomic data

#### Olink proteomic data

Protein biomarkers related to cardiovascular health and inflammation were measured in blood plasma using 3 Olink panels: Cardiovascular II, Cardiovascular III, and Inflammation (Olink Proseek Multiplex Kits). Olink technology is based on a proximity extension assay and measures normalized protein expression (NPX). NPX is a relative quantification of protein and is reported on a log2 scale. Intensity normalization was performed by Olink for each panel.

#### Protein missingness

A total of 276 proteins were initially available for analysis across the 3 panels (Cardiovascular II, Cardiovascular III, and Inflammation). Proteins with >50% values below the limit of detection (LOD) were not included in the analyses (*n* = 24) (Supplemental Figure 2). For the remaining proteins, values below the LOD were replaced with the LOD for that protein. Some proteins (*n* = 10) were measured in >1 panel (e.g., Cardiovascular II and Inflammation) (Supplemental Figure 2); for redundant proteins, the panel with the higher LOD and fewer missing values was retained. A total of 242 proteins remained for subsequent analysis.

#### Sample quality control

Samples that failed Olink quality control (e.g., deviated >0.3 NPX from the median value of internal controls) and/or had no data were excluded from analysis. This accounted for only ∼1.3% (*n* = 2281) of total observations (*n* = 171,336; 242 proteins × 708 samples) in the dataset. Principal component (PC) analysis of NPX values revealed 2 outlier samples in PC1, each >3 SDs from the mean (Supplemental Figure 3). These samples were both collected at 6 mo and were excluded.

### Statistical analysis

Given established sex differences in body composition (e.g., males typically have more LST and less FM), the following analyses were stratified by sex [23]. The original DIETFITS trial indicated that weight loss and body fat percentage loss did not differ significantly between diets after 12 mo [1]; thus, we pooled the 2 treatment arms in the following analyses.

#### Investigating changes in LST after 6 mo of a diet-based intervention

Paired t-tests were used to compare changes in body composition measures (total mass, LST, aLST, FM, and percentage predicted LST) from baseline to 6 mo, at a 5% significance level. Total body and appendicular regions were assessed. Percentage predicted LST was log-transformed prior to statistical tests to ensure normality. The proportions of LST and FM lost out of total mass were not normally distributed and are reported as medians with interquartile ranges (IQR).

#### Sensitivity analyses to examine the impact of diet and TEE

We conducted sensitivity analyses to examine whether changes in body composition varied by diet or TEE. T-tests were used to compare changes in LST, appendicular LST, and total mass between diets. Proportions of LST and appendicular LST lost on each diet were compared using Wilcoxon rank-sum tests. Linear regression models were fit with LST change as the dependent variable, with TEE (the mean of 3-mo and 6-mo measurements) and diet as predictors. All statistical tests were 2-sided, and an α level of 0.05 was used to determine statistical significance.

#### Exploring proteomic predictors of change in LST

##### Residual analysis

The change in LST was regressed against the change in FM. Residuals were extracted and used as the outcome variable (e.g., LST residuals). Baseline protein concentrations (Protein_Baseline_) and changes in protein concentrations (ΔProtein = Protein_6Mo_ - Protein_Baseline_) were examined as predictors of LST residuals in separate univariate models: LST residuals ∼ Protein_Baseline_ and LST residuals ∼ ΔProtein. Each unique protein was tested in an independent linear model. Significant predictors were identified using the Benjamini–Hochberg procedure to control the false discovery rate (FDR) at 5% [24], separately for the baseline and protein change models. Multivariable models adjusted for diet, age, height, and race/ethnicity were also implemented and were the focus of analyses. Given that a minimal change in appendicular LST was observed in the DXA data, we modeled LST that had greater change from baseline to 6 mo.

##### Elastic net

To identify a smaller set of features that predicted LST residuals, an elastic net model with all proteomic changes (ΔProtein), diet, age, height, race (Black, Asian, or Other), and ethnicity (Hispanic), was created with the *glmnet* package in R [22]. 10-fold cross-validation with *cv.glmnet* was performed using all available data to select the hyperparameters λ and α that minimized the mean squared error [22]. To enhance robustness, elastic net models were fit to 1000 bootstrap samples from the full dataset, using the λ and α determined by cross-validation. Predictors were reported if they appeared in >60% of 1000 bootstrapped models, along with the median and IQR of their β coefficients. The 60% threshold was chosen to be stringent while still allowing for exploratory analysis.

##### Gene set enrichment analysis

Proteins in our dataset were mapped to their corresponding genes using the UniProt database (https://www.uniprot.org/). Hallmark pathways, obtained from the Molecular Signatures Database (MSigDB), comprise 50 gene sets that encapsulate established biological states and processes and were utilized for gene set enrichment analysis [25]. Gene set enrichment analysis was conducted with the *fgsea* package in R using t-statistics from multivariable models [26].

##### PC regression

As a complementary analysis, we performed PC regression to identify any patterns of protein expression associated with LST residuals. Protein concentrations were standardized after removing missing values. PC analysis with varimax rotation was performed using the *psych* package in R [27]. A scree plot was used to determine the number of PCs to retain. The retained PCs were extracted and used as independent variables in a linear regression model to predict LST residuals. If any PCs were significant predictors of LST residuals (*p* < 0.05), the factor loadings were examined to identify the top proteins contributing to that component. All analyses were performed with R version 4.2.3 (R Core Team, 2024).

## Results

### Participant demographics

Of the 609 participants in the DIETFITS study, 374 individuals had DXA data at both baseline and 6 mo (Table 1). Participants had a mean age ± SD of 39.4 ± 6.7 y and were predominately female (60.7%), White (72.7%), and non-Hispanic (78.1%). Both female and male participants had obesity (average BMI > 30) at baseline. Approximately half of the participants were randomly assigned to an HLC diet (51.9%) or an HLF diet (48.1%).

**TABLE 1:** Demographics of DIETFITS participants with DXA measurements at baseline and at 6 mo

Baseline demographics	Cohort with baseline and 6-mo DXA (*n* = 374)
	Mean ± SD or *n* (%)
Age (y)	39.4 ± 6.7
Sex	
Female	227 (60.7)
Male	147 (39.3)
Race	
White	272 (72.7)
Other	47 (12.6)
Asian	34 (9.1)
Black/African American	14 (3.7)
American Indian/Alaskan	2 (0.5)
Native Hawaiian/Pacific Islander	2 (0.5)
NA	3 (0.8)
Ethnicity	
Non-Hispanic	292 (78.1)
Hispanic	80 (21.4)
NA	2 (0.5)
Diet	
Healthy low-carbohydrate	194 (51.9)
Healthy low-fat	180 (48.1)
BMI (kg/m^2^)	
Female	32.1 ± 3.2
Male	32.5 ± 3.3
Total mass^1^ (kg)	
Female	86.8 ± 11.5
Male	101.6 ± 12.8
Total lean soft tissue (kg)	
Female	49.0 ± 6.1
Male	67.8 ± 7.4
Total fat mass (kg)	
Female	35.4 ± 6.8
Male	30.8 ± 7.6
Appendicular lean soft tissue^2^ (kg)	
Female	21.1 ± 3.1
Male	30.6 ± 3.7
Appendicular fat mass^2^ (kg)	
Female	17.1 ± 3.9
Male	12.1 ± 3.4
Body fat (%)	
Female	40.6 ± 4.0
Male	30.1 ± 4.8

Abbreviations: DIETFITS, Diet Intervention Examining The Factors Interacting with Treatment Success; DXA, dual-energy X-ray absorptiometry; NA, not available.

1 Includes lean soft tissue, fat mass, and bone mineral content measured by DXA.

2 Arms and legs only; excludes head and trunk regions.

### Changes in LST after 6 mo of diet-based weight loss

After 6 mo of diet-based weight loss, both sexes lost ∼6.9% of their initial body mass. This corresponds to a change in total mass of −5.9 kg (95% confidence interval [CI]: −6.51, −5.29) in females and −7.18 kg (95% CI: −8.2, −6.16) in males (Table 2). The majority of this loss could be attributed to FM, whereas LST accounted for only 26.7% and 32.5% of total mass loss in females and males, respectively.

**TABLE 2:** Total body: change in LST after 6 mo of diet-based weight loss

Measure	Female (*n* = 227)		Male (*n* = 147)	
	Mean difference (95% CI),^1^ kg	Proportion (%) of total mass loss, median (IQR)	Mean difference (95% CI),^1^ kg	Proportion (%) of total mass loss, median (IQR)
Total mass	−5.9 (−6.51, −5.29)	—	−7.18 (−8.2, −6.16)	—
FM	−4.28 (−4.75, −3.81)	73.5 (63.3, 87.5)	−4.92 (−5.64, −4.2)	67.0 (53.0, 80.0)
LST	−1.61 (−1.84, −1.38)	26.7 (12.4, 36.6)	−2.26 (−2.65, −1.86)	32.5 (20.3, 46.5)
aLST^2^	−0.86 (−1.06, −0.66)	13.6 (−3.0, 25.45)	−1.39 (−1.72, −1.06)	20.7 (6.2, 37.2)

Abbreviations: aLST, adjusted lean soft tissue; CI, confidence interval; FM, fat mass; LST, lean soft tissue.

1 All *P* values for mean differences from paired t-tests were significant (*P* < 0.001).

2 aLST is an adjusted dual-energy X-ray absorptiometry measure that excludes fat-free adipose tissue.

After adjusting for FFAT, changes in aLST accounted for <25% of total mass loss in both sexes (Table 2, Supplemental Figure 4). This corresponds to absolute changes in aLST of only −0.86 kg (95% CI: −1.06, −0.66) in females and −1.39 kg (95% CI: −1.72, −1.06) in males across the entire body.

Changes in appendicular (arms and legs) mass accounted for <50% of the total mass loss (Table 3), which indicates mass was primarily lost from other body regions (e.g., trunk). The majority of appendicular mass loss was from FM rather than LST. In fact, losses of appendicular LST—a surrogate for skeletal muscle—were minimal and accounted for only ∼12.8% of total mass loss in both sexes.

**TABLE 3:** Appendicular region: change in LST after 6 mo of diet-based weight loss

Measure	Female (*n* = 227)		Male (*n* = 147)	
	Mean difference (95% CI),^1^ kg	Proportion (%) of total mass loss, median (IQR)	Mean difference (95% CI),^1^ kg	Proportion (%) of total mass loss, median (IQR)
Appendicular mass	−2.9 (−3.2, −2.61)	49.1 (41.9, 58.1)	−2.9 (−3.32, −2.49)	40.4 (34.0, 45.9)
Appendicular FM	−2.09 (−2.32, −1.87)	36.0 (28.6, 45.5)	−1.87 (−2.15, −1.6)	25.4 (19.8, 34.5)
Appendicular LST	−0.8 (−0.92, −0.69)	12.8 (5.5, 18.8)	−1.02 (−1.22, −0.83)	12.8 (3.2, 21.9)
Appendicular aLST^2^	−0.43 (−0.54, −0.33)	6.7 (−2.0, 14.3)	−0.69 (−0.86, −0.53)	8.2 (−2.3, 18.1)

Abbreviations: aLST, adjusted lean soft tissue; CI, confidence interval; FM, fat mass; LST, lean soft tissue.

1 All *P* values for mean differences from paired t-tests were significant (*p* < 0.001).

2 aLST is an adjusted dual-energy X-ray absorptiometry measure that excludes fat-free adipose tissue.

After correcting for FFAT, changes in appendicular aLST were attenuated and accounted for only 6.7% and 8.2% of total mass loss in females and males, respectively. This corresponds to small absolute changes in appendicular aLST of −0.43 kg (95% CI: −0.54, −0.33) and −0.69 kg (95% CI: −0.86, −0.53) for females and males, respectively.

A sensitivity analysis was conducted to evaluate whether diet (HLC or HLF) influenced changes in body composition. Absolute losses in total mass, LST, and appendicular LST were significantly greater on HLC in males, but not in females (Supplemental Figure 5). However, relative losses (e.g., the proportions of LST and appendicular LST lost from total mass) were not significantly different between diets (Supplemental Figure 6). We also assessed whether physical activity levels, measured by TEE (kcal/kg/d), impacted changes in LST (Supplemental Tables 3 and 4). However, TEE was not significantly associated with change in LST.

### Percentage predicted appendicular LST

To evaluate whether LST was proportional to body size, we calculated percentage predicted LST, where a value of 100% indicates that an individual’s observed mass is equal to their predicted mass. The DIETFITS-derived models we used to predict appendicular LST are shown in Table 4.

**TABLE 4:** DIETFITS-derived models for appendicular LST

DIETFITS-derived appendicular LST models	
Female	ln(Appendicular LST) = 0.33 + 0.60∗ln(Appendicular Mass) + 1.03∗ln(Height) + 0.02(I_Black_) + 0.02(I_RaceOther_)^1^
Male	ln(Appendicular LST) = 0.75 + 0.63∗ln(Appendicular Mass) + 0.48∗ln(Height) + 0.05(I_Black_) - 0.004(I_Asian_)

Abbreviations: DIETFITS, Diet Intervention Examining The Factors Interacting with Treatment Success; LST, lean soft tissue.

1 “RaceOther” is defined as not American Indian/Alaskan, Asian, Black/African American, Native Hawaiian/Pacific Islander, or White.

**FIGURE 4:**
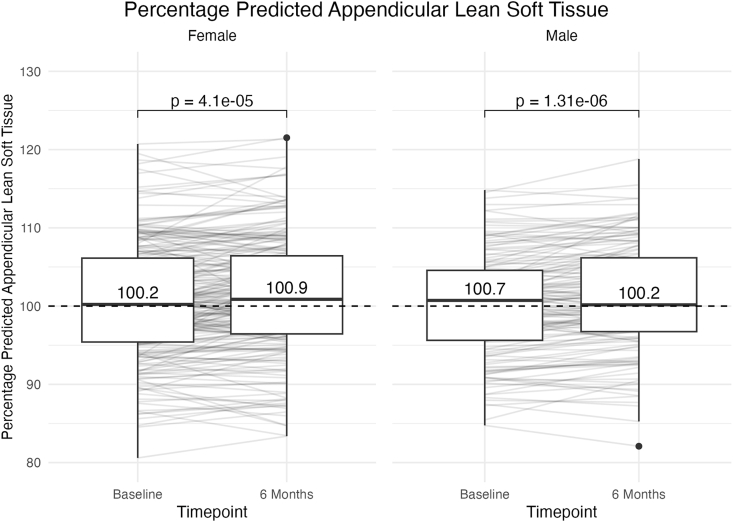
Percentage predicted appendicular lean soft tissue (LST) at baseline compared with 6 mo of diet-based weight loss. The median values and IQRs for percentage predicted appendicular LST (observed LST/predicted LST) are displayed for females and males. A value >100% indicates more LST than predicted for body size. Even after weight loss, individuals had more appendicular LST than expected (>100%). Paired t-tests, accounting for individual differences, revealed significant increases from baseline to 6 mo, indicating that both sexes had “proportionally more” appendicular LST relative to body size after diet-based weight loss. Paired t-tests were conducted on log-transformed data.

Percentage predicted appendicular LST was adequate at all time points (>100%) and did not decline with weight loss (Figure 4). In fact, percentage predicted appendicular LST increased from baseline to 6 mo in paired analyses for both sexes (*p* < 0.001) (Table 5). This indicates that individuals had “proportionally more” appendicular LST relative to body size after diet-based weight loss. Percentage predicted total body LST also increased at 6 mo (*p* < 0.001; Supplemental Tables 5 and 6, Supplemental Figure 7).

**TABLE 5:** Percentage predicted appendicular LST: mean difference and 95% CIs

Measure	Sex	Mean ± SD at baseline (%)	Mean ± SD at 6 mo (%)	Mean difference (95% CI)^1^
Percentage predicted appendicular LST	Female	100.5 ± 7.5	101.4 ± 7.6	0.82 (0.44, 1.21)
	Male	100.2 ± 6.6	101.1 ± 6.9	0.92 (0.57, 1.28)

Percentage predicted appendicular LST was log-transformed to ensure normality prior to statistical tests.

Abbreviations: CI, confidence interval; LST, lean soft tissue.

1 Mean difference is calculated as 6 mo − baseline, where positive values indicate an increase at 6 mo. All *P* values for mean differences from paired t-tests were significant (*P* < 0.001); exact *P* values are shown in Figure 4.

### Longitudinal changes in LST compared with FM

**FIGURE 5:**
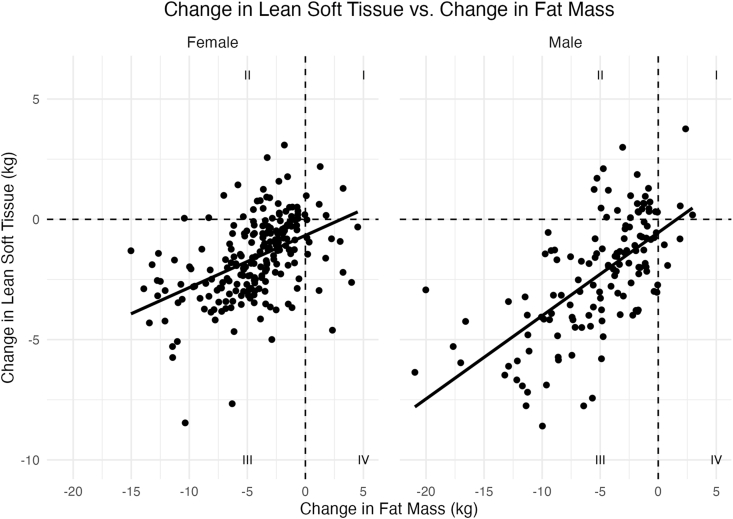
Change in lean soft tissue (LST) compared with change in fat mass (FM). The trend line (black) shows the expected change in LST for a given change in FM in the Diet Intervention Examining The Factors Interacting with Treatment Success cohort (*n* = 374). In our cohort, individuals with positive residuals (above the regression line) retained or gained more LST than expected for their given change in FM. Individuals with negative residuals (below the regression line) lost more LST than expected for their given change in FM. Residuals for LST are calculated as observed LST − predicted change in LST.

Despite modest changes in LST after 6 mo of weight loss, some individuals experienced greater LST changes relative to FM than others. The relationship between change in LST compared with change in FM for females and males is shown in Figure 5. Most participants simultaneously lost both FM and LST (quadrant III). However, several participants managed to gain LST while losing FM (quadrant II), or vice versa (quadrant IV). LST residuals were extracted from the models in Figure 5 and used as the dependent variable in the proteomic models discussed in the next section.

### Proteomic features associated with LST

**FIGURE 6:**
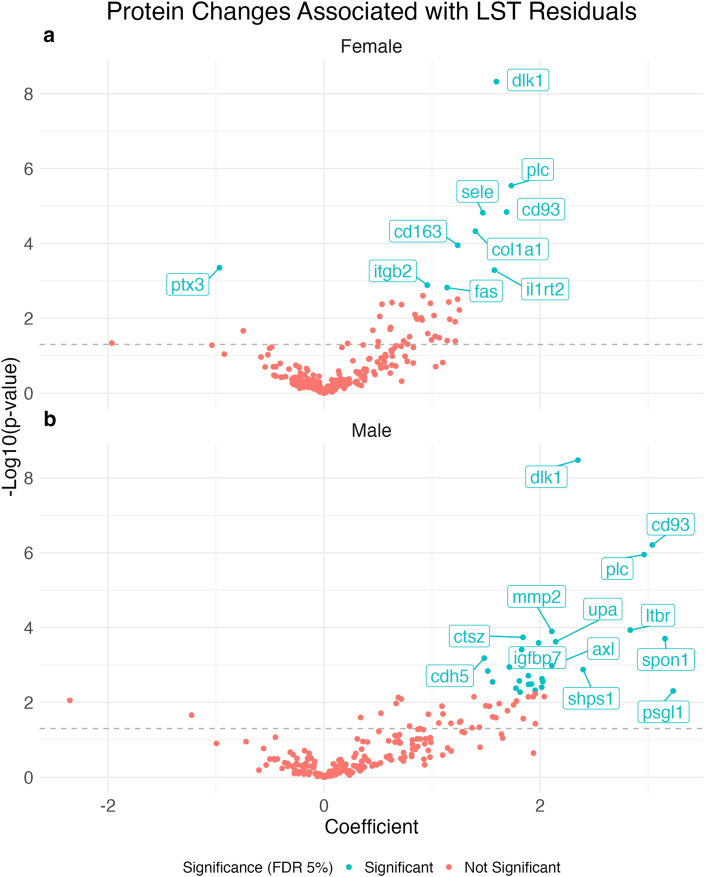
Volcano plot of protein changes associated with lean soft tissue (LST) residuals. Protein changes associated with LST residuals are shown for (A) female and (B) male participants. Change in protein delta homolog 1 (DLK1) was the top predictor of LST residuals in females and males based on *P* values. This volcano plot is based on multivariable models; the results of univariate models are shown in Supplemental Figure 9. Uniprot IDs for the significant proteins are provided in Supplemental Tables 7 and 8. FDR, false discovery rate.

No protein concentrations at baseline alone predicted LST residuals (Supplemental Figure 8). However, protein changes (ΔProtein = Protein_6Mo_ − Protein_Baseline_) were strong predictors of LST residuals, with 10 protein changes showing significant associations in females and 27 in males (Figure 6, Supplemental Tables 7 and 8). Protein delta homolog 1 (DLK1), a known inhibitor of adipogenesis, was significant and had the smallest *P* value in both sexes. Changes in complement component C1q receptor (CD93) and Perlecan (PLC) were also significantly associated with LST residuals in both sexes. The majority of significant proteins had positive coefficients, indicating that increases in protein concentrations were associated with increases in LST residuals (i.e., the change in LST was adjusted for the change in FM).

**FIGURE 7:**
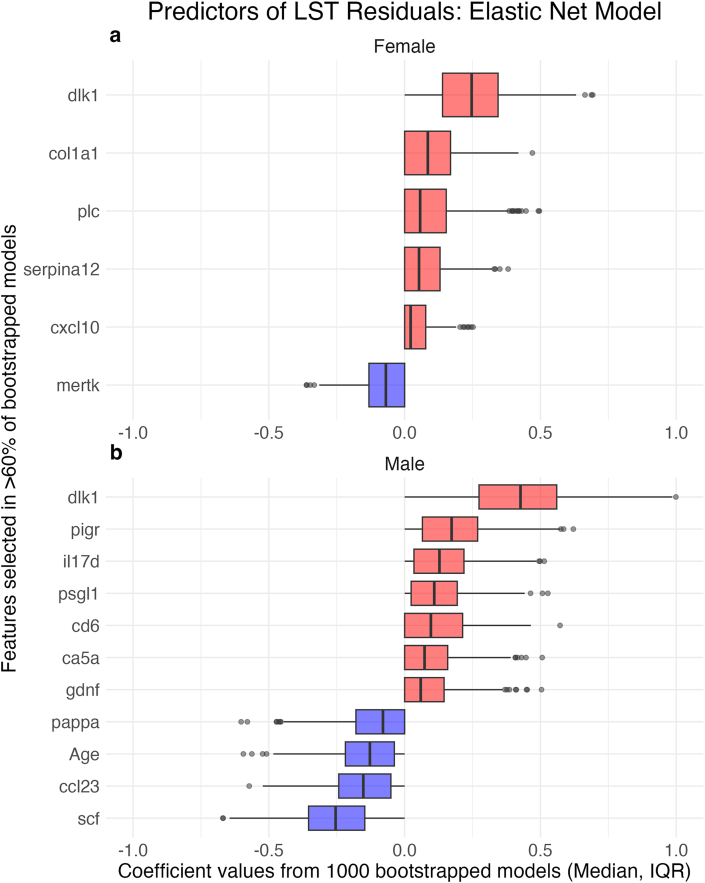
Elastic net model: set of protein changes associated with lean soft tissue (LST) residuals. We used an elastic net model to identify the minimum set of protein changes that predicted LST residuals. After selecting the optimal α and λ parameters using 10-fold cross-validation, 1000 bootstrapped elastic net models were analyzed. Features selected in >60% of models, as well as their coefficients (median and IQR), are displayed for (A) female and (B) male participants. Uniprot IDs for the proteins are provided in Supplemental Table 9.

To identify the minimum set of protein changes that predicted LST residuals, we used an elastic net model with bootstrapping. After selecting the optimal α and λ parameters using 10-fold cross-validation, 1000 bootstrapped elastic net models were generated. Features selected in >60% of models, along with their coefficients (median with IQR), are displayed in Figure 7 and Supplemental Table 9. These results highlight DLK1 as the top protein (e.g., largest median coefficient), which is consistent with the multivariable model results in Figure 6, where DLK1 was also the top protein.

### Pathways and higher-order biology associated with change in LST

Gene set enrichment analysis was carried out after running multivariable models. None of the Hallmark pathways from MSigDB—50 gene sets capturing established biological states and processes—were significantly enriched (5% FDR) (Supplemental Figure 10).

We also used PC regression to identify patterns of protein expression associated with LST residuals. In females, we identified 3 significant PCs associated with LST residuals (Supplemental Tables 10–12, Supplemental Figure 11); these were dominated by proteins related to immune function (PC1), vascular markers (PC3), and metabolic markers (PC8). In males, we similarly found 2 significant PCs (Supplemental Figure 12, Supplemental Tables 13 and 14) related to immune function (PC1) and metabolic markers (PC7). Interestingly, the PCs for females and males representing “metabolic markers” included known markers related to energy balance and lipid metabolism (e.g., leptin [LEP] and proprotein convertase subtilisin/kexin type 9 [PCSK9]), in addition to DLK1—our top protein hit from linear models.

## Discussion

In this study, we conducted a secondary analysis of the DIETFITS study (*n* = 374), one of the largest cohorts to complete a diet-based weight-loss intervention. Our key findings include the following: *1*) changes in LST and appendicular LST—a surrogate for skeletal muscle mass—were minimal after 6 mo of weight loss; *2*) these changes were further attenuated after adjusting for FFAT; and *3*) appendicular LST did not decrease disproportionately after 6 mo of weight loss. In an exploratory analysis, we also identified several key proteins, including DLK1, that are associated with changes in LST after adjusting for changes in FM.

Previous diet-based studies in individuals with overweight or obesity suggest that LST accounts for 20% to 30% of total weight loss, with the majority of weight loss being from FM [7,[28], [29], [30]]. This is consistent with our findings: LST losses accounted for 26.7% and 32.5% of total weight loss in females and males, respectively. Many diet-based studies do not distinguish LST from appendicular LST [[28], [29], [30]], even though appendicular LST is a better surrogate for skeletal muscle mass [16]. Diet-based weight-loss studies reporting appendicular LST show modest losses (∼1 kg for 5%–10% body weight loss) but have small sample sizes (*n* < 100) [[31], [32], [33], [34], [35]]. Our study replicates these findings for appendicular LST in a significantly larger cohort (*n* = 374), in which we observed −0.80 kg (95% CI: −0.92, −0.69) and −1.02 kg (95% CI: −1.22, −0.83) in females and males, with 6.9% total mass loss in both sexes, respectively. Our study strongly reinforces that changes in appendicular LST, a surrogate for skeletal muscle mass, are minimal after a diet-based weight-loss intervention.

We observed that changes in LST and appendicular LST were attenuated after adjusting for FFAT. DXA includes FFAT, a component of adipose tissue, in its LST measurement (Figure 3). This can lead to overestimation of LST losses, especially when large amounts of adipose tissue are lost [11]. Abe et al. [11] introduced the concept of adjusting LST for FFAT (Figure 3) as a method to improve DXA-derived LST measures. After adjusting for FFAT, Abe et al. [11] observed minimal LST loss or even increases in LST after weight loss in 2 smaller cohorts (*n* = 38 and *n* = 141) [11]. Our study applied these corrections to a significantly larger cohort and demonstrated that changes in appendicular LST were further reduced to −0.43 kg (95% CI: −0.54, −0.33) and −0.69 kg (95% CI: −0.86, −0.53) in females and males, and accounted for only 6.7% and 8.2% of total mass loss, respectively. These changes are smaller than those reported in many previous studies [31,32,35]. Notably, our cohort is younger than many diet-based weight-loss cohorts; this may have been a protective factor against LST loss. However, we expect that applying FFAT adjustments in other adult cohorts would similarly attenuate estimated LST losses.

We conducted sensitivity analyses to examine the impact of diet and physical activity on LST change. Males lost significantly more LST, appendicular LST, and total mass on HLC. However, the proportion of LST and appendicular LST lost did not differ between diets. At this time, our data suggest that HLC, compared with HLF, does not lead to meaningful differences in weight-loss composition, consistent with the original DIETFITS trial [1]. We also did not find significant associations between physical activity, measured by TEE, and LST change. Future studies with more sensitive measurements may be better suited to quantify the impact of physical activity on LST changes.

Recently, clinical researchers have questioned whether reductions in LST during weight loss are clinically relevant [36]. Inspired by percentage predicted metrics already used in clinical practice (e.g., ppVO_2_), percentage predicted LST compares an individual’s observed LST with the expected value based on their body size and demographics. We found that percentage predicted appendicular LST increased and remained >100% at 6 mo, indicating that participants had greater amounts of appendicular LST than expected for their body size and demographics, even after weight loss. These findings suggest that LST loss could be a natural adaptation to diet-based weight loss. Individuals with obesity generally have more muscle mass than healthy-weight individuals [37]; after weight loss, they may not require the same absolute amount of LST to support their reduced body size.

In this study, we also identified novel proteins, such as DLK1, associated with LST retention during weight loss. Existing studies have found several proteins associated with weight loss but do not isolate those associated with LST changes, independent of FM changes [[12], [13], [14], [15]]. In both females and males, DLK1 emerged as the top predictor of LST residuals after adjusting for FM. DLK1, also known as preadipocyte factor 1, inhibits adipocyte differentiation [38]. Previous research has shown that DLK1-knockout mice exhibit accelerated adiposity, whereas mice overexpressing DLK1 in vivo show a decrease in FM [39,40]. These previous findings, and our observation that increased DLK1 was associated with increased LST, suggest that DLK1 may be a compelling biomarker for LST retention during weight loss to explore in future research.

Several other proteomic features were associated with LST residuals, including changes in PLC and CD93. PLC is found in the extracellular matrix of tissues (e.g., muscle and adipose) and plays a role in tissue structure and remodeling [41]. CD93 is known for its role in angiogenesis and is expressed in the endothelium, the inner cellular lining of blood vessels [42]. Other top proteins that were associated with LST residuals included structural proteins related to alpha-1 type I collagen (COL1A1) in females and insulin-like growth factor-binding protein 7 (IGFBP7) in males. Moving forward, proteins related to inhibition of adipogenesis (DLK1), tissue structure (PLC and COL1A1), and angiogenesis and cell growth (CD93 and IGFBP7) may serve as useful biomarkers for studying LST preservation.

Our study has several strengths. First, we applied an updated methodology—focusing on appendicular LST, adjusting for FFAT, and evaluating LST relative to body size—to more rigorously evaluate the claim that weight loss leads to LST loss. In this study, we applied this methodology to one of the largest longitudinal diet-based weight-loss cohorts—nearly 400 individuals—and found that diet-based weight loss did not result in disproportionate losses in LST. In addition, we identified several protein biomarkers, including DLK1, associated with LST retention during weight loss.

There are several limitations within our study. First, DXA was used to assess changes in body composition; MRI provides a more accurate quantification of changes in skeletal muscle. However, this study provided us with the opportunity to showcase a simple correction for FFAT (Figure 3) that enables researchers to estimate tissue-level changes from DXA. Second, our analysis was limited to 242 plasma proteins associated with cardiovascular health and inflammation. Other features not included in our dataset—additional proteins, metabolites, transcripts, or cytokines in blood plasma, or measures from other tissues (e.g., muscle and adipose)—may provide stronger explanations for changes in LST. Lastly, in future studies, collecting tissue biopsy samples to assess muscle quality after weight loss may be even more important than assessing muscle quantity.

Importantly, our results differ from findings in pharmacologic trials of new weight-loss drugs, such as glucagon-like peptide-1 receptor agonists (GLP-1RAs), including semaglutide, tirzepatide, and liraglutide. Several clinical trials have reported that GLP-1RAs can lead to proportionally larger reductions in LST (e.g., 40% to 60% of total weight loss), although absolute reductions in LST are often small [36,43]. Direct comparisons between diet-based and pharmacologic interventions are needed. In the meantime, our results suggest that diet-based weight loss does not lead to disproportionate LST losses.

### Conclusion

In a secondary analysis of one of the largest diet-based weight-loss cohorts, we found that changes in appendicular LST—a surrogate for skeletal muscle mass—were minimal, accounting for <10% of total mass loss in both sexes after adjusting for FFAT. Diet-based weight loss did not lead to disproportionate losses of LST or appendicular LST, suggesting it is a safe strategy for reducing body weight while preserving musculoskeletal health. Proteomic features, including DLK1, a known inhibitor of adipogenesis, may provide mechanistic insight into LST retention during weight loss.

## Author contributions

The authors’ responsibilities were as follows – CDG: conducted the Diet Intervention Examining The Factors Interacting with Treatment Success (DIETFITS) clinical trial; FH, AKR: conceptualized this DIETFITS substudy; AKR: analyzed the data; AKR, FH: wrote the manuscript; DJP, BH, CPW, MPS: provided consultation on study design and feedback on the manuscript; and all authors: read and approved the final manuscript.

## Data availability

Data described in the manuscript, code book, and analytic code will be made available upon reasonable request.

## Funding

The authors reported no funding received for this study.

## Conflict of Interest

MPS is a cofounder and scientific advisor of Crosshair Therapeutics, Exposomics, Filtricine, Fodsel, iollo, InVu Health, January AI, Marble Therapeutics, Mirvie, Next Thought AI, Orange Street Ventures, Personalis, Protos Biologics, Qbio, RTHM, and SensOmics; scientific advisor of Abbratech, Applied Cognition, Enovone, Jupiter Therapeutics, M3 Helium, Mitrix, Neuvivo, Onza, Sigil Biosciences, TranscribeGlass, WndrHLTH, and Yuvan Research; cofounder of NiMo Therapeutics; investor and scientific advisor of R42 and Swaza; and is also an investor in Repair Biotechnologies. All other authors report no conflicts of interest.
