# Supplementary material for: Diet-based weight-loss intervention is not associated with a meaningful change in lean soft tissue
Source: Am J Clin Nutr. 2026 Feb 23;123(5):101251. doi: 10.1016/j.ajcnut.2026.101251 (PMC13197904; doi:10.1016/j.ajcnut.2026.101251)
Supplement: Multimedia component 1 [file mmc1.docx]

# **Supplementary Materials**

# **Supplementary Table 1.** Calculating Adjusted Lean Soft Tissue (aLST) from DXA

| **Step** | **Formula** | **Description** |
| --- | --- | --- |
| 1. | AT = FM + FFAT  AT = FM/0.85 | **Basic relationship.** Adipose tissue (AT) is composed of fat mass (FM) and fat-free adipose tissue (FFAT).  AT is estimated to be composed of approximately 85% FM, 15% FFAT. |
| 2. | FFAT = AT - FM  FFAT = (FM/0.85) - FM  **FFAT = 0.176FM** | **Estimate fat-free adipose tissue (FFAT).** |
| 3. | aLST = LST - FFAT  **aLST = LST - 0.176FM** | **Estimate adjusted lean soft tissue (aLST)**: lean soft tissue (LST) minus fat-free adipose tissue (FFAT). |

## **Supplementary Table 2.** Demographics of DIETFITS Participants with Dual energy X-ray absorptiometry (DXA) Measurements at Baseline and/or 6 Months

| Baseline Demographics | Mean (SD) or n (%) | |
| --- | --- | --- |
|  | Cohort with Baseline DXA (n=466)^1^ | Cohort with Baseline and 6 Month DXA (n=374) |
| Age (years) | 39.1 (6.7) | 39.4 (6.7) |
| Sex  Female  Male | 276 (59.2)  190 (40.8) | 227 (60.7)  147 (39.3) |
| Race  White  Other  Asian  Black/African American  American Indian/Alaskan  Native Hawaiian/Pacific Islander  NA | 330 (70.8)  59 (12.7)  44 (9.4)  19 (4.1)  3 (0.6)  3 (0.6)  8 (1.7) | 272 (72.7)  47 (12.6)  34 (9.1)  14 (3.7)  2 (0.5)  2 (0.5)  3 (0.8) |
| Ethnicity  Non-Hispanic  Hispanic  NA | 360 (77.3)  104 (22.3)  2 (0.4) | 292 (78.1)  80 (21.4)  2 (0.5) |
| Diet  Healthy Low-Carbohydrate  Healthy Low-Fat | 238 (51.1)  228 (48.9) | 194 (51.9)  180 (48.1) |
| BMI (kg/m^2^)  Female  Male | 32.3 (3.2)  32.5 (3.2) | 32.1 (3.2)  32.5 (3.3) |
| Total Mass (kg)^2^  Female  Male | 87.2 (11.6)  102.0 (12.6) | 86.8 (11.5)  101.6 (12.8) |
| Total Lean Soft Tissue (kg)  Female  Male | 49.2 (6.1)  68.1 (7.4) | 49.0 (6.1)  67.8 (7.4) |
| Total Fat Mass (kg)  Female  Male | 35.7 (6.9)  31.0 (7.3) | 35.4 (6.8)  30.8 (7.6) |
| Appendicular Lean Soft Tissue (kg)^3^  Female  Male | 21.2 (3.1)  30.7 (3.7) | 21.1 (3.1)  30.6 (3.7) |
| Appendicular Fat Mass (kg)^3^  Female  Male | 17.2 (3.9)  12.2 (3.3) | 17.1 (3.9)  12.1 (3.4) |
| Body Fat (%)  Female  Male | 40.7 (4.0)  30.1 (4.6) | 40.6 (4.0)  30.1 (4.8) |

^1^The 466 participants with DXA data at baseline were used to generate DIETFITS Appendicular Lean Soft Tissue (LST) prediction models.

^2^Includes Lean Soft Tissue, Fat Mass, and Bone Mineral Content; measured by dual-energy X-ray absorptiometry (DXA).

^3^Arms and legs only; excludes head and trunk regions.

## **Supplementary Figure 1.** Performance of NHANES LST models (**a-b**), DIETFITS LST models **(c-d)**, and DIETFITS Appendicular LST models (**e-f**). Performance on the test set (20% of female or male baseline DXA data) was high, with R^2^ >.70 for nearly all models. Predicted LST or Predicted Appendicular LST values were used in the denominator of our Percentage Predicted LST metric (observed LST/predicted LST).


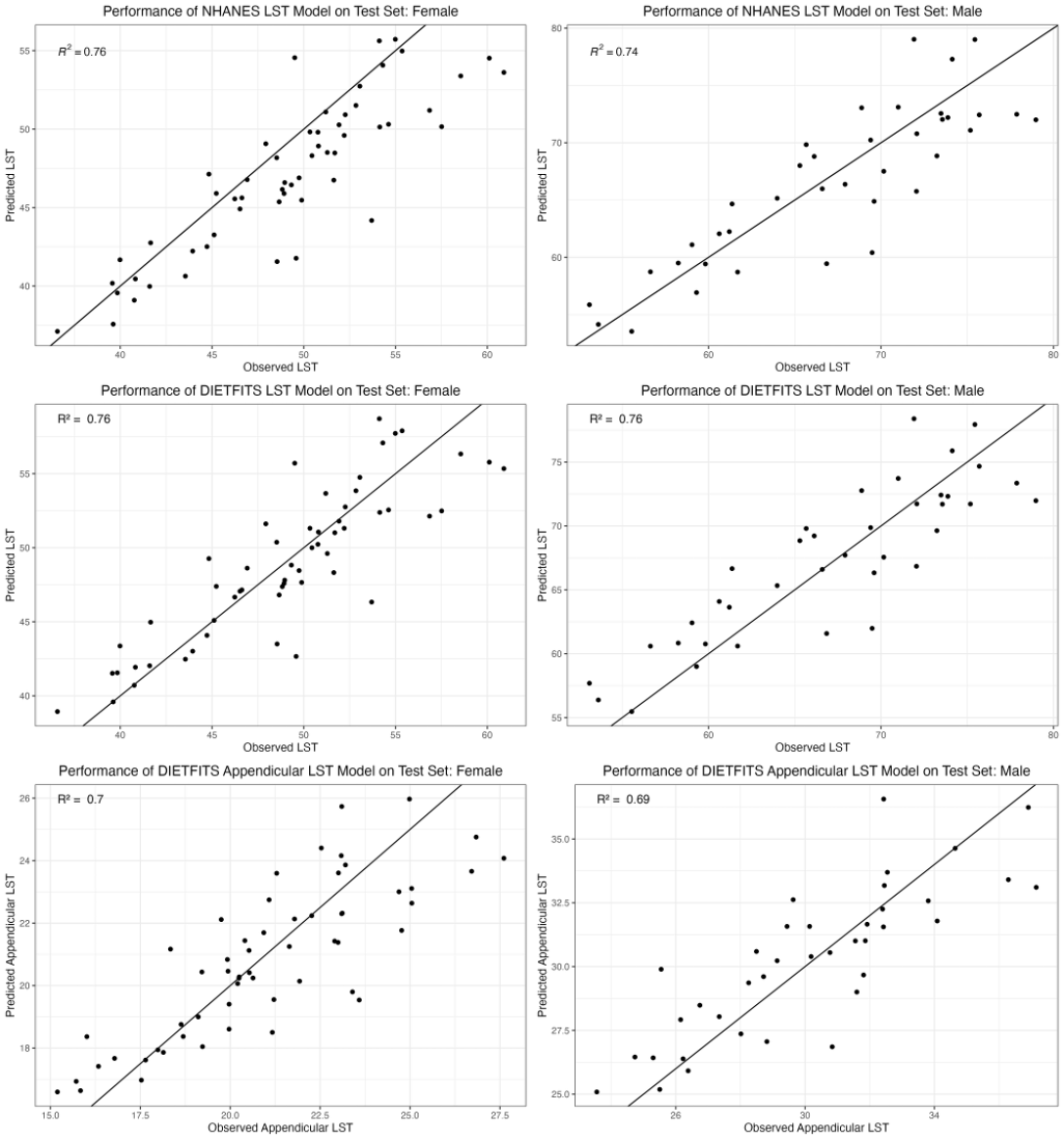


##

## **Supplementary Figure 2**. Olink Data Cleaning and Quality Control.

276 proteins

252 proteins

>50% below limit of detection (n=24)

242 proteins

Duplicated on >=2 panels (n=10)*

*Chose protein on panel with less missingness for subsequent analyses.

1. The 24 proteins excluded for >50% observations below limit of detection included SLAMF7, BNP, NT-pro BNP, MCP-3, IL-17C, IL-17A, IL-20RA, IL-2RB, IL-1 alpha, IL-2, TSLP, IL-10RA, IL-22RA1, IL-24, IL-13, ARTN, TNF, IL-20, IL-33, INF-gamma, IL-4, LIF, NRTN, IL-5.
2. The 10 duplicated proteins included OPG, uPA, IL-6, CXCL1, CCL3, MCP-1, SCF, IL-18, FGF-23, FGF-21.

## **Supplementary Figure 3.** Principal Component Analysis: Baseline and 6 Month Olink Data


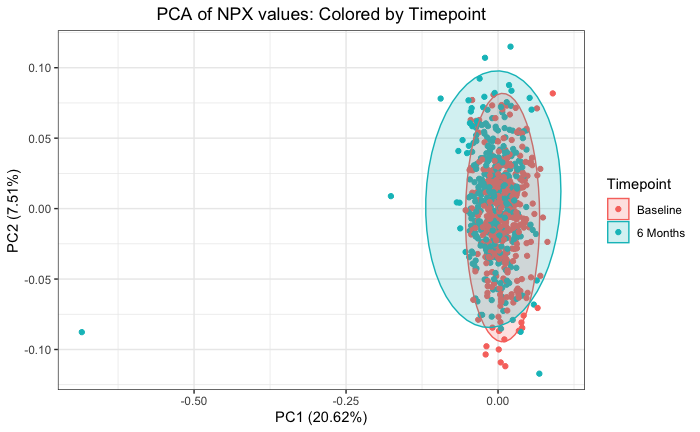


## **Supplementary Figure 4.** Proportion of Total Weight Loss Derived from Lean Soft Tissue vs. Fat Mass


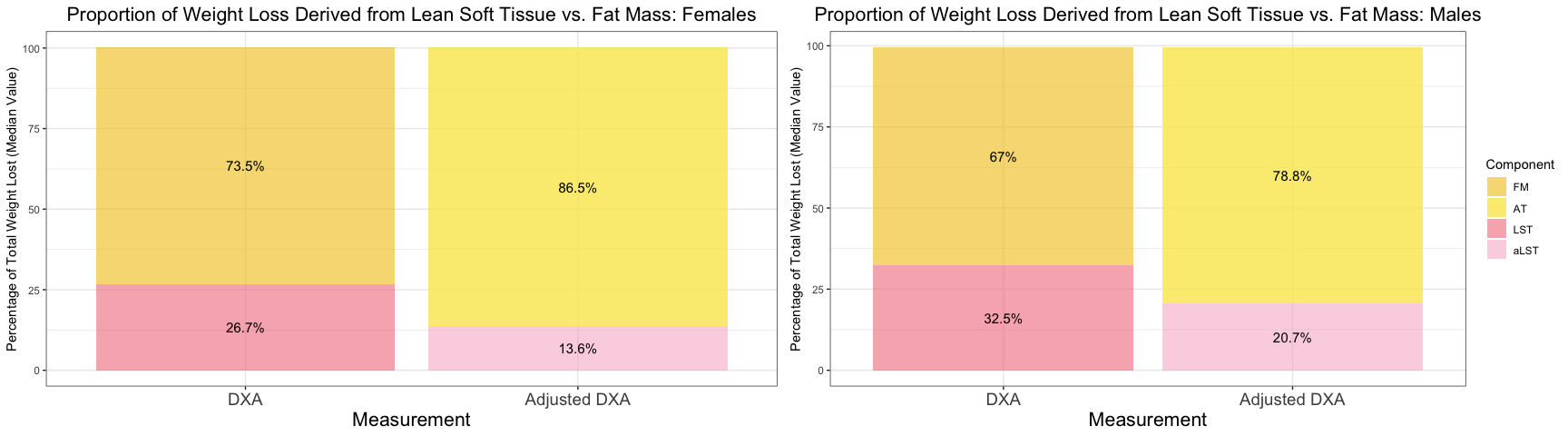


**Supplementary Figure 5.** Absolute Change in Body Composition Metrics by Diet


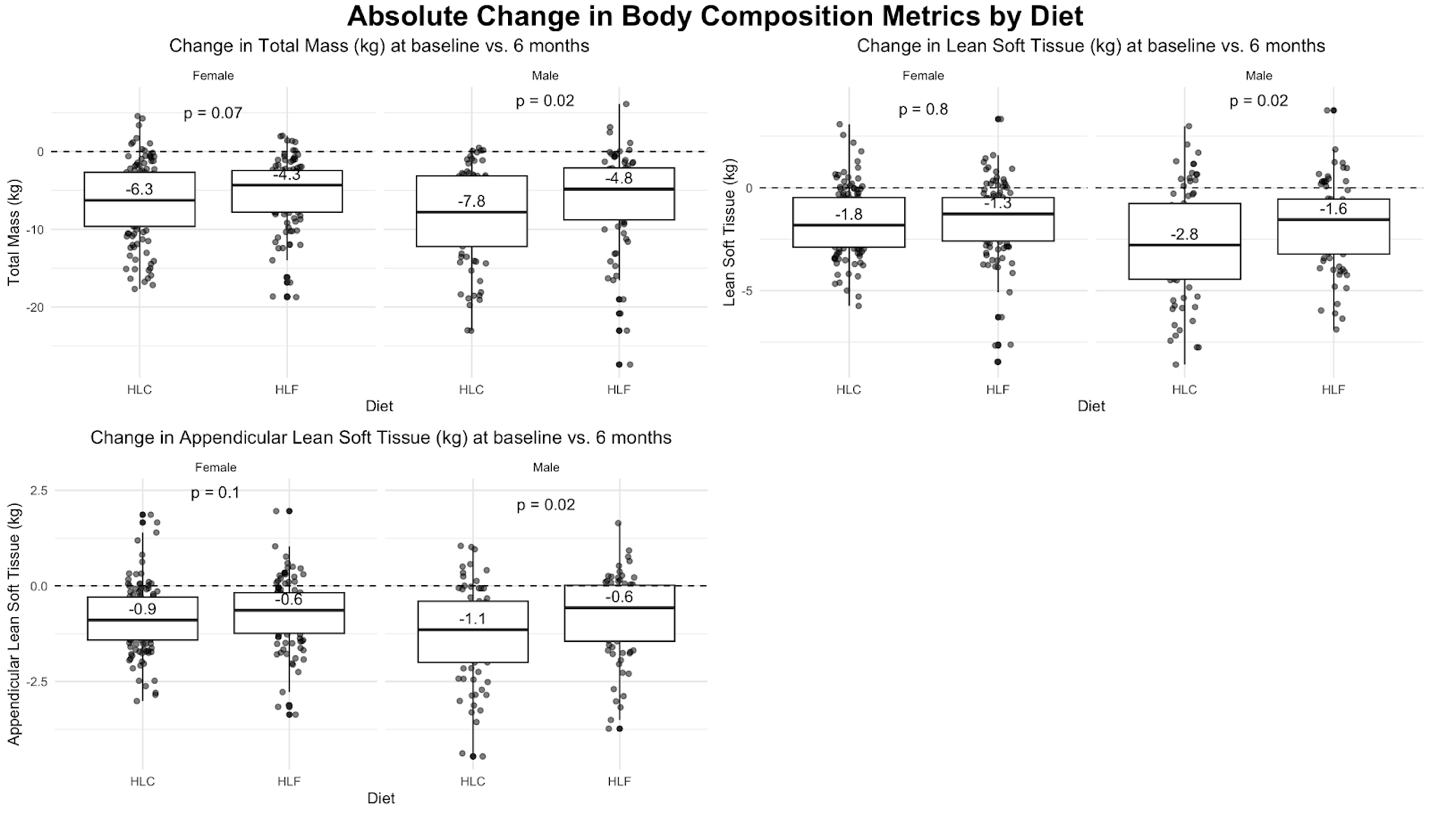


**Supplementary Figure 6.** Similar Proportion of Lean Soft Tissue (LST) Loss on Diets


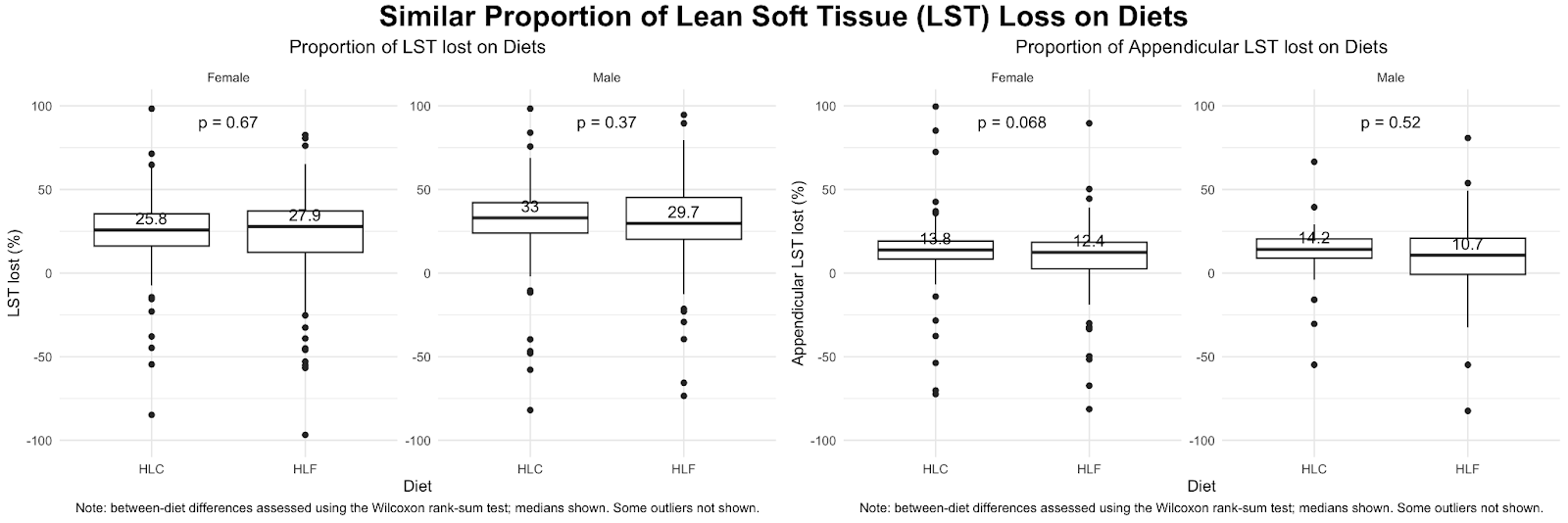


**Supplementary Table 3.** Linear Models to Estimate Impact of Average Total Energy Expenditure (TEE) on Change in Lean Soft Tissue: Females

| Model | Term | Estimate | Standard Error | Statistic | p-value |
| --- | --- | --- | --- | --- | --- |
| LST | Intercept | 0.58 | 2.20 | 0.27 | 0.79 |
|  | DietHLF | 0.05 | 0.24 | 0.20 | 0.84 |
|  | Average TEE (kcal/kg/day)^1^ | -0.07 | 0.07 | -1.00 | 0.32 |
| Appendicular LST | Intercept | 0.65 | 1.10 | 0.59 | 0.55 |
|  | DietHLF | 0.19 | 0.12 | 1.54 | 0.12 |
|  | Average TEE (kcal/kg/day)^1^ | -0.05 | 0.03 | -1.39 | 0.17 |

^1^Calculated as the mean of 3 Month and 6 Month survey measurements

*p<0.05

HLF = Healthy Low-Fat

**Supplementary Table 4.** Linear Model to Estimate Impact of Average Total Energy Expenditure (TEE) on Change in Lean Soft Tissue: Males

| Model | Term | Estimate | Standard Error | Statistic | p-value |
| --- | --- | --- | --- | --- | --- |
| LST | Intercept | -0.83 | 2.84 | -0.29 | 0.77 |
|  | DietHLF | 0.97 | 0.41 | 2.40 | 0.02* |
|  | Average TEE (kcal/kg/day)^1^ | -0.06 | 0.09 | -0.68 | 0.50 |
| Appendicular LST | Intercept | 0.26 | 1.37 | 0.19 | 0.85 |
|  | DietHLF | 0.49 | 0.20 | 2.52 | 0.01* |
|  | Average TEE (kcal/kg/day)^1^ | -0.05 | 0.04 | -1.13 | 0.26 |

^1^Calculated as the mean of 3 Month and 6 Month survey measurements

*p<0.05

HLF = Healthy Low-Fat

## **Supplementary Table 5.** DIETFITS-derived LST models

| **DIETFITS-derived LST models** | |
| --- | --- |
| **LST** |  |
| Female | ln(LST) = 0.68 + 0.62*ln(Total Mass) + 0.91*ln(Height) + 0.02(I_RaceOther_)^a^ |
| Male | ln(LST) = 1.17 + 0.59*ln(Total Mass) + 0.60*ln(Height) + 0.04(I_Black_) - 0.02(I_Asian_) - 0.003(I_RaceOther_)^a^ |

^a^Race Other is defined as **not** American Indian/Alaskan, Asian, Black/African American, Native Hawaiian/Pacific Islander, or White.

## **Supplementary Table 6.** Mean Difference and 95% CIs for Percentage Predicted Lean Soft Tissue from Baseline to 6 Months

| Reference | Measure | Gender |  | Mean at Baseline (%) | Mean at 6 Months (%) | Mean Difference^1^ (%) | 95% CI |
| --- | --- | --- | --- | --- | --- | --- | --- |
| NHANES | Percentage Predicted LST | Male |  | 101.2 | 103.1 | 1.84 | (1.41, 2.27) |
|  |  | Female |  | 104.3 | 105.5 | 1.22 | (0.86, 1.57) |
| DIETFITS | Percentage Predicted LST | Male |  | 100.0 | 101.0 | 0.98 | (0.61, 1.36) |
|  |  | Female |  | 100.3 | 101.4 | 1.12 | (0.76, 1.47) |

^1^Mean Difference is calculated as 6 Month - Baseline, where positive values indicate an increase at 6 months. All p-values for mean differences from paired t-tests were significant (p<0.001); exact p-values are shown in **Supplementary Figure 7**. Percentage predicted LST was log-transformed to ensure normality prior to statistical tests.

**Supplementary Figure 7.** Assessing LST relative to body size: total body percentage predicted LST using (a) NHANES and (b) DIETFITS models at baseline vs. 6 months. Paired t-tests were conducted on log-transformed data.

**
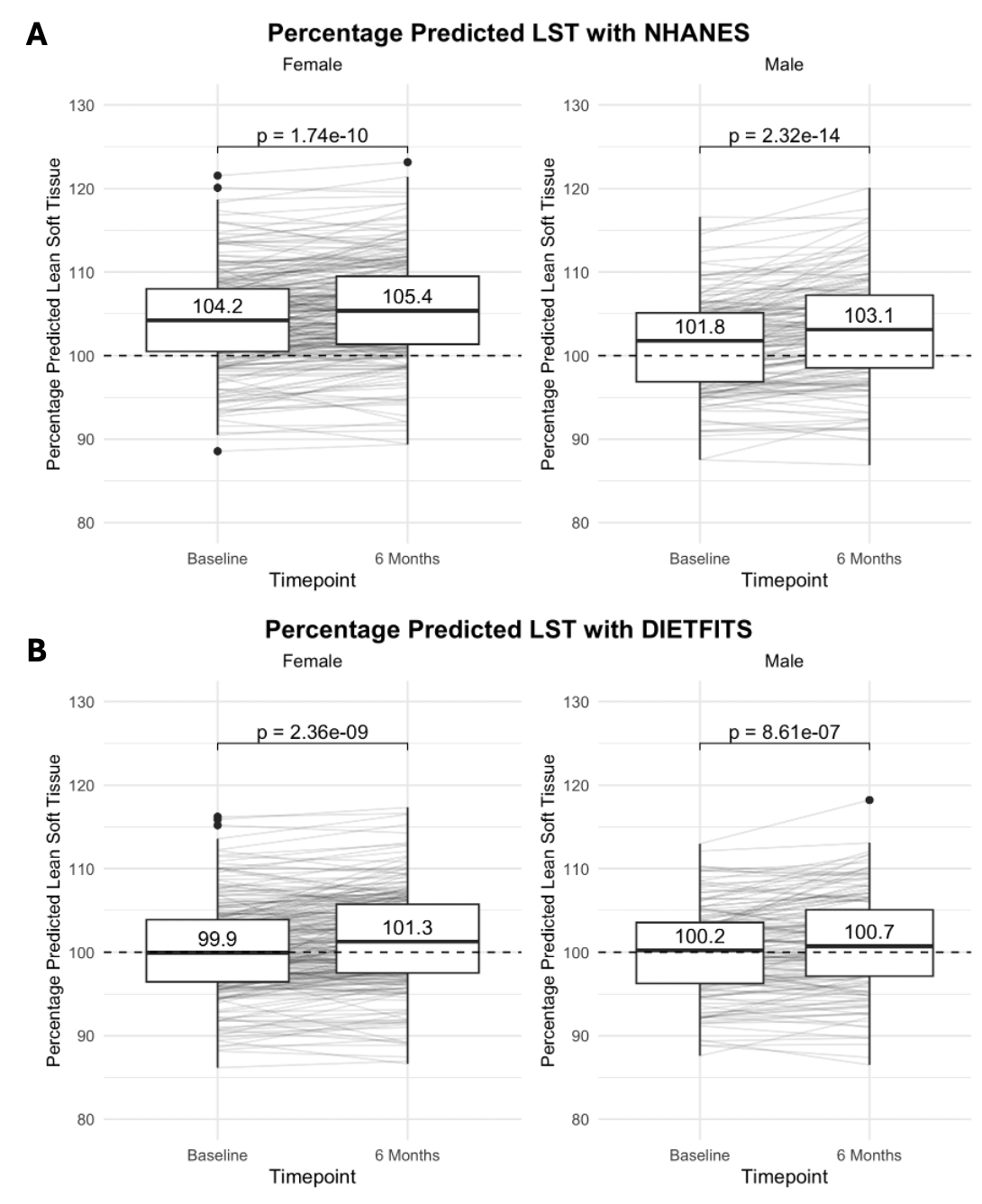
**

## **Supplementary Figure 8.** Baseline Proteins Associated with LST Residuals in a) Female and b) Males (Multivariable Models)


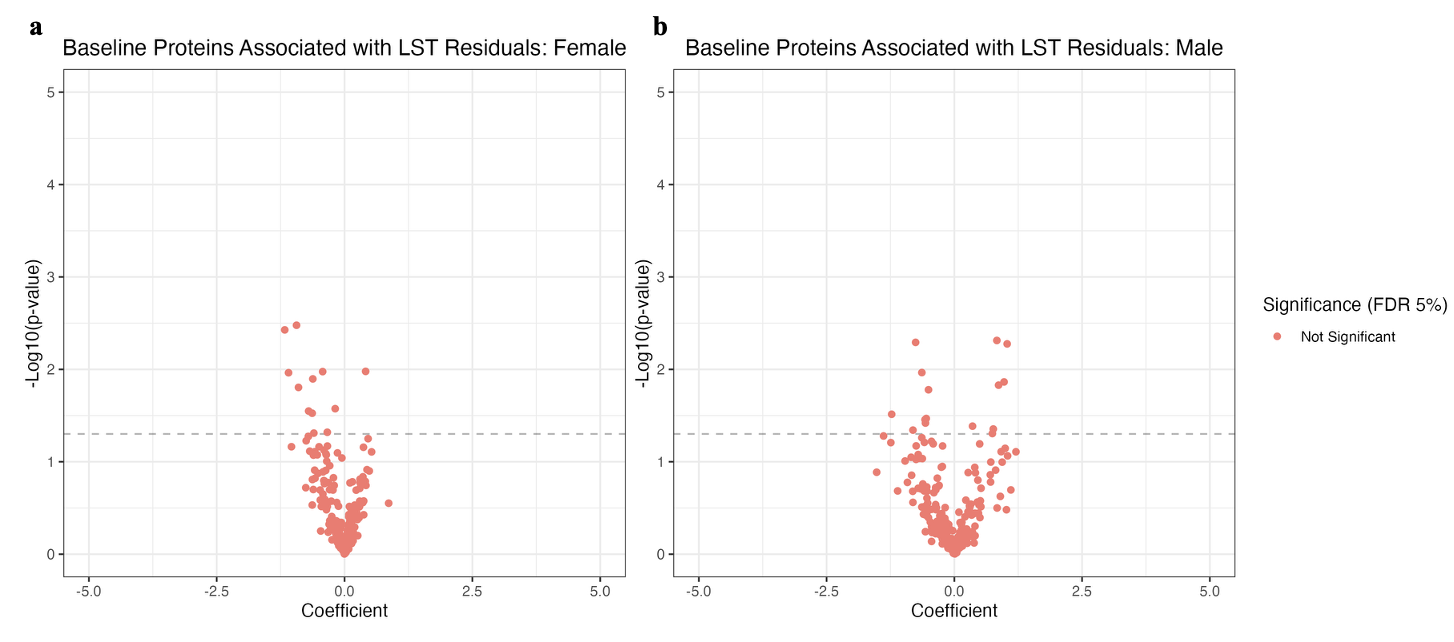


## **Supplementary Figure 9.** Volcano Plot of **a-b)** Baseline Proteins and **c-d**) Protein Changes from Month 0 to Month 6 Associated with LST Residuals in Females and Males (Univariate Models)


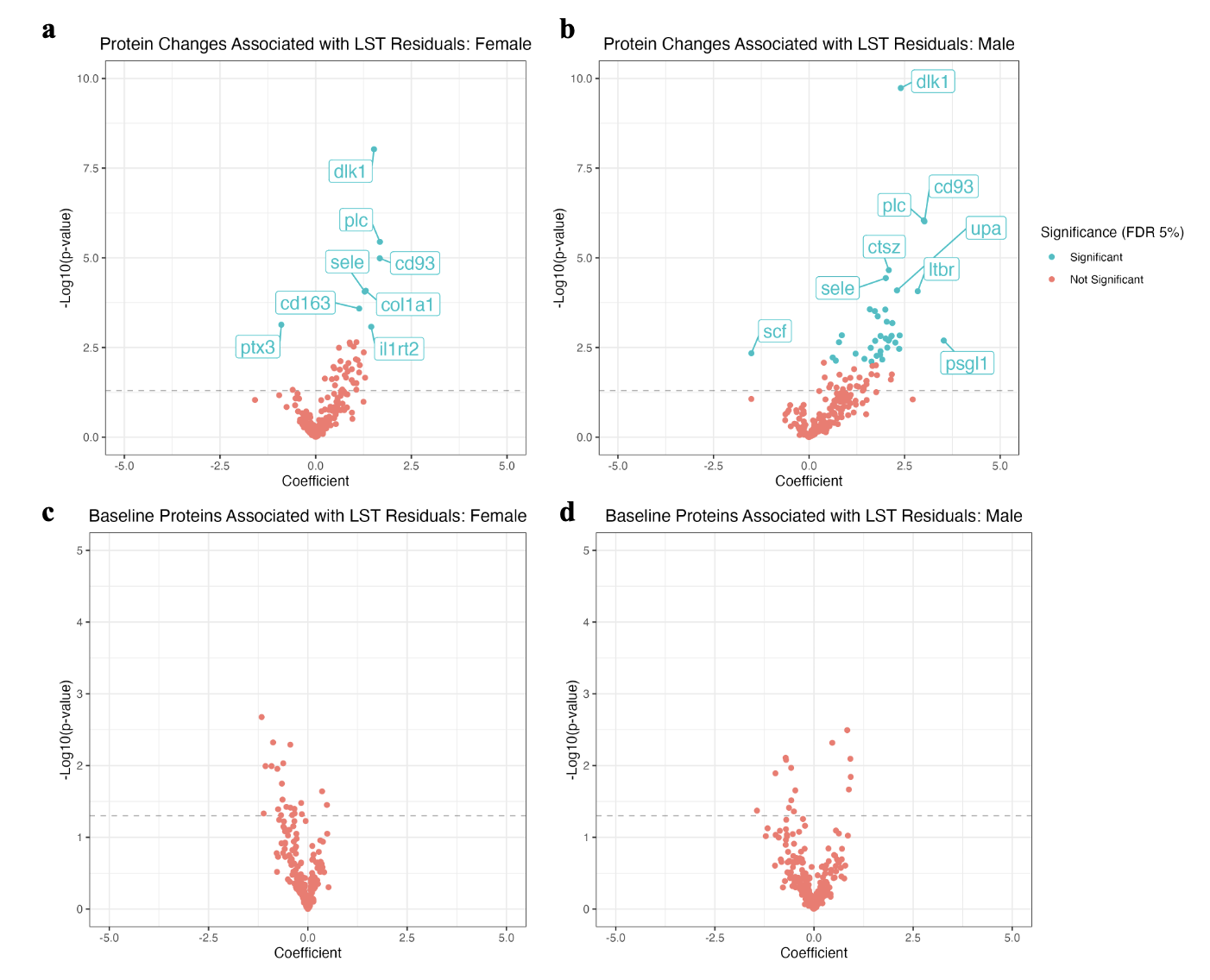


## **Supplementary Table 7.** Significant Protein Changes (n=10) Associated with LST Residuals from Multivariable Models: Females (5% FDR)

| **Protein Name** | **Protein (short)** | **Coefficient** | **Adjusted P-Value (5% FDR)** | **Uniprot ID** |
| --- | --- | --- | --- | --- |
| Protein delta homolog 1 | dlk1 | 1.6 | 1e-06 | P80370 |
| Perlecan | plc | 1.73 | 0.000344 | P98160 |
| E-selectin | sele | 1.47 | 0.000911 | P16581 |
| Complement component C1q receptor | cd93 | 1.69 | 0.000911 | Q9NPY3 |
| Collagen alpha-1 chain | col1a1 | 1.4 | 0.002276 | P02452 |
| Scavenger receptor cysteine-rich type 1 protein M130 | cd163 | 1.24 | 0.004504 | Q86VB7 |
| Pentraxin-related protein PTX3 | ptx3 | -0.97 | 0.01534 | P26022 |
| Interleukin-1 receptor type 2 | il1rt2 | 1.58 | 0.015603 | P27930 |
| Integrin beta-2 | itgb2 | 0.96 | 0.034909 | P05107 |
| Tumor necrosis factor receptor superfamily member 6 | fas | 1.14 | 0.036649 | P25445 |

## **Supplementary Table 8**. Significant Protein Changes (n=27) Associated with LST Residuals from Multivariable Models: Males (5% FDR)

| **Protein Name** | **Protein (short)** | **Coefficient** | **Adjusted P-Value (5% FDR)** | **Uniprot ID** |
| --- | --- | --- | --- | --- |
| Protein delta homolog 1 | dlk1 | 2.35 | 1e-06 | P80370 |
| Complement component C1q receptor | cd93 | 3.04 | 7.5e-05 | Q9NPY3 |
| Perlecan | plc | 2.96 | 9e-05 | P98160 |
| Lymphotoxin-beta receptor | ltbr | 2.83 | 0.0061 | P36941 |
| Matrix metalloproteinase-2 | mmp2 | 2.11 | 0.0061 | P08253 |
| Spondin-1 | spon1 | 3.16 | 0.006811 | Q9HCB6 |
| Cathepsin Z | ctsz | 1.84 | 0.006811 | Q9UBR2 |
| Urokinase-type plasminogen activator | upa | 2.14 | 0.006883 | P00749 |
| Insulin-like growth factor-binding protein 7 | igfbp7 | 1.99 | 0.006883 | Q16270 |
| E-selectin | sele | 1.83 | 0.009367 | P16581 |
| Cadherin-5 | cdh5 | 1.48 | 0.014417 | P33151 |
| Tyrosine-protein kinase receptor UFO | axl | 2.11 | 0.020969 | P30530 |
| Scavenger receptor cysteine-rich type 1 protein M130 | cd163 | 1.72 | 0.02103 | Q86VB7 |
| Tyrosine-protein phosphatase non-receptor type substrate 1 | shps1 | 2.4 | 0.022832 | P78324 |
| Integrin beta-2 | itgb2 | 1.52 | 0.023381 | P05107 |
| Fms-related tyrosine kinase 3 ligand | flt3l | 1.89 | 0.029168 | P49771 |
| Contactin-1 | cntn1 | 2.02 | 0.033213 | Q12860 |
| CD166 antigen | alcam | 2.03 | 0.034239 | Q13740 |
| C-C motif chemokine 15 | ccl15 | 1.56 | 0.034239 | Q16663 |
| Glial cell line-derived neurotrophic factor | gdnf | 1.81 | 0.034239 | P39905 |
| Osteoprotegerin | opg | 1.92 | 0.036249 | O00300 |
| Tumor necrosis factor receptor 1 | tnfr1 | 1.89 | 0.036249 | P19438 |
| Interleukin-17D | il17d | 2.02 | 0.041249 | Q8TAD2 |
| T cell surface glycoprotein CD6 isoform | cd6 | 1.78 | 0.042104 | Q8WWJ7 |
| Ephrin type-B receptor 4 | ephb4 | 1.95 | 0.045805 | P54760 |
| P-selectin glycoprotein ligand 1 | psgl1 | 3.23 | 0.045871 | Q14242 |
| Tumor necrosis factor receptor superfamily member 10C | tnfrsf10c | 1.81 | 0.047521 | O14798 |

## **Supplementary Table 9**. Predictors of LST Residuals: Elastic Net Models

| **Sex** | **Protein Name** | **Protein (short)** | **Uniprot** |
| --- | --- | --- | --- |
| Female | Protein delta homolog 1 | dlk1 | P80370 |
|  | Collagen alpha-1 chain | col1a1 | P02452 |
|  | Perlecan | plc | P98160 |
|  | Serpin A12 | serpina12 | Q8IW75 |
|  | C-X-C motif chemokine 10 | cxcl10 | P02778 |
|  | Tyrosine-protein kinase Mer | mertk | Q12866 |
| Male^1^ | Protein delta homolog 1 | dlk1 | P80370 |
|  | Polymeric immunoglobulin receptor | pigr | P01833 |
|  | Interleukin-17D | il17d | Q8TAD2 |
|  | P-selectin glycoprotein ligand 1 | psgl1 | Q14242 |
|  | T cell surface glycoprotein CD6 isoform | cd6 | Q8WWJ7 |
|  | Carbonic anhydrase 5A, mitochondrial | ca5a | P35218 |
|  | Glial cell line-derived neurotrophic factor | gdnf | P39905 |
|  | Pappalysin-1 | pappa | Q13219 |
|  | C-C motif chemokine 23 | ccl23 | P55773 |
|  | Stem cell factor | scf | P21583 |

^1^Note: Age was also a predictor identified by elastic net for male LST residuals but is omitted.

## **Supplementary Figure 10.** Results of Gene Set Enrichment Analysis.

a) Gene Set Enrichment Analysis for protein changes associated with LST residuals: Females


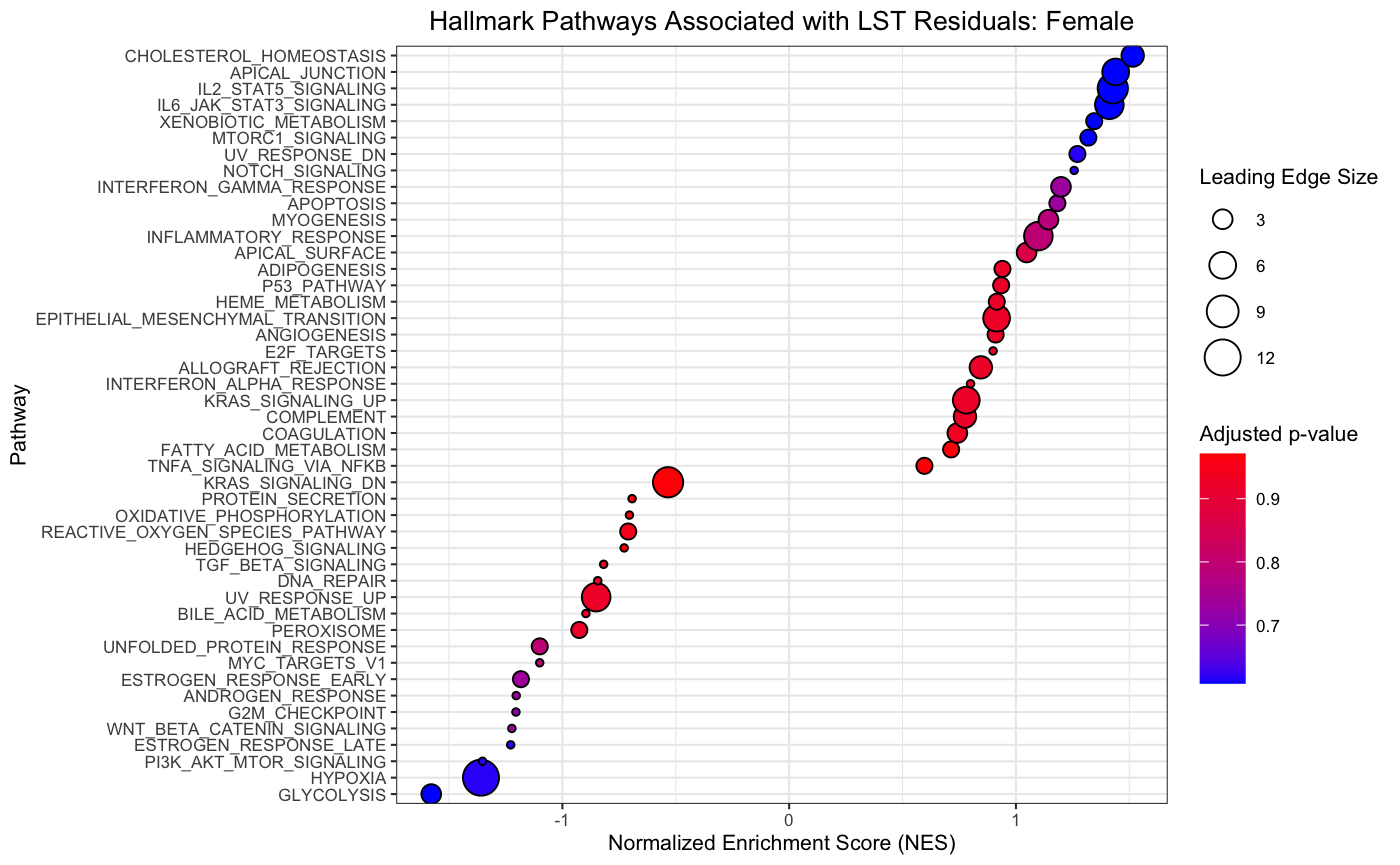


b) Gene Set Enrichment Analysis for protein changes associated with LST residuals: Males


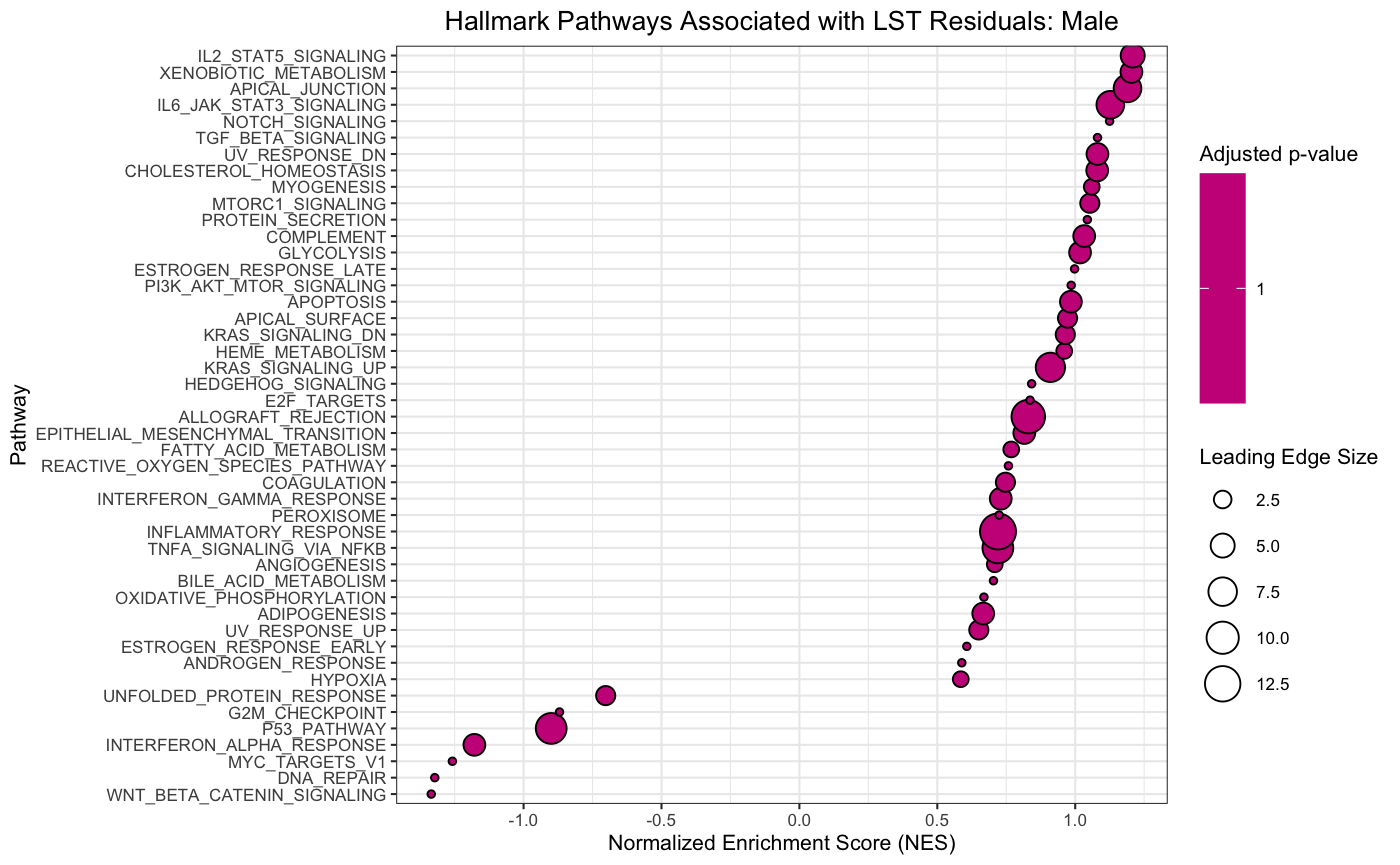


**Supplementary Figure 11**. Principal Component Regression: Predicting LST Residuals in Females


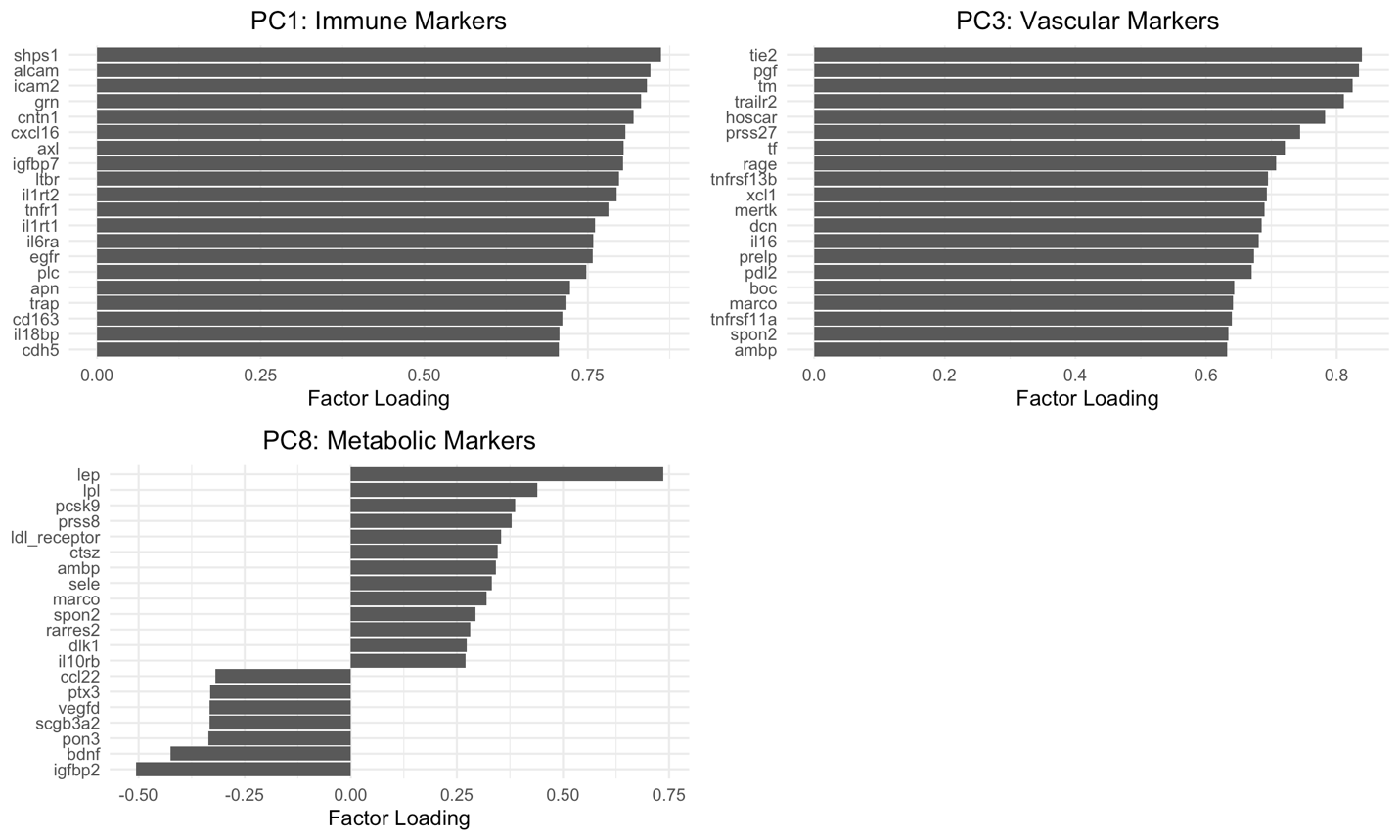


**Supplementary Figure 12.** Principal Component Regression: Predicting LST Residuals in Males
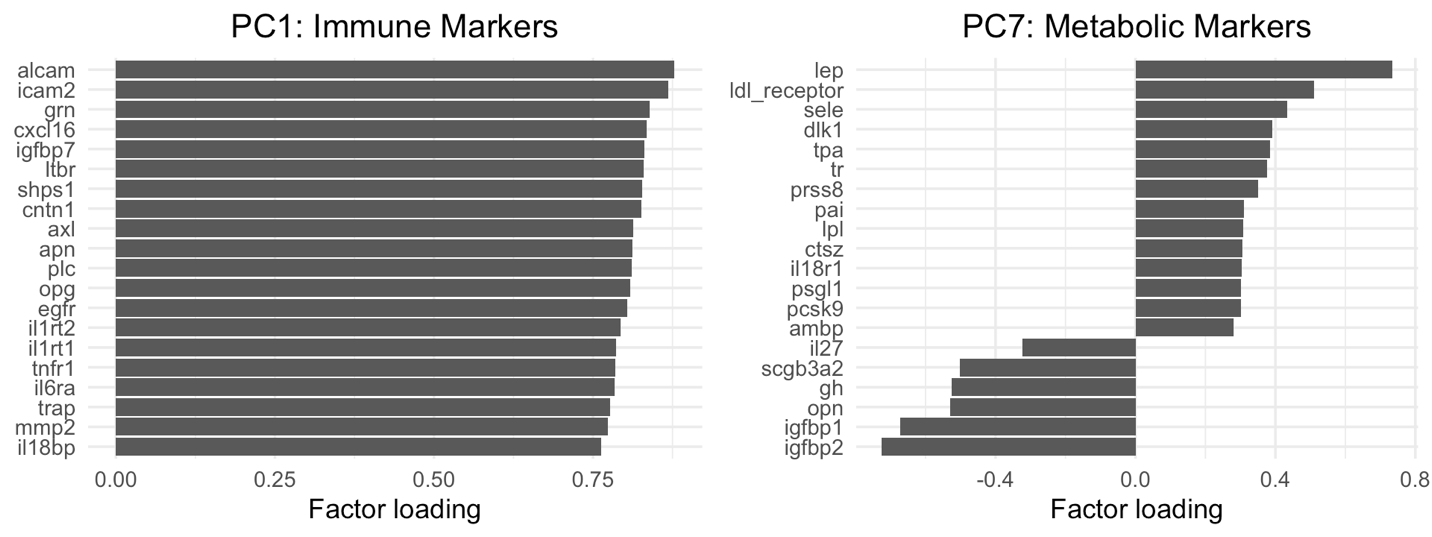


## **Supplementary Table 10**. PC1 Top 20 Proteins: Females

| **Protein Name** | **Protein (short)** | **Uniprot** |
| --- | --- | --- |
| Tyrosine-protein phosphatase non-receptor type substrate 1 | shps1 | P78324 |
| CD166 antigen | alcam | Q13740 |
| Intercellular adhesion molecule 2 | icam2 | P13598 |
| Granulins | grn | P28799 |
| Contactin-1 | cntn1 | Q12860 |
| C-X-C motif chemokine 16 | cxcl16 | Q9H2A7 |
| Tyrosine-protein kinase receptor UFO | axl | P30530 |
| Insulin-like growth factor-binding protein 7 | igfbp7 | Q16270 |
| Lymphotoxin-beta receptor | ltbr | P36941 |
| Interleukin-1 receptor type 2 | il1rt2 | P27930 |
| Tumor necrosis factor receptor 1 | tnfr1 | P19438 |
| Interleukin-1 receptor type 1 | il1rt1 | P14778 |
| Interleukin-6 receptor subunit alpha | il6ra | P08887 |
| Epidermal growth factor receptor | egfr | P00533 |
| Perlecan | plc | P98160 |
| Aminopeptidase N | apn | P15144 |
| Tartrate-resistant acid phosphatase type 5 | trap | P13686 |
| Scavenger receptor cysteine-rich type 1 protein M130 | cd163 | Q86VB7 |
| Interleukin-18-binding protein | il18bp | O95998 |
| Cadherin-5 | cdh5 | P33151 |

## **Supplementary Table 11**. PC3 Top 20 Proteins: Females

| **Protein Name** | **Protein (short)** | **Uniprot** |
| --- | --- | --- |
| Angiopoietin-1 receptor | tie2 | Q02763 |
| Placenta growth factor | pgf | P49763 |
| Thrombomodulin | tm | P07204 |
| TNF-related apoptosis-inducing ligand receptor 2 | trailr2 | O14763 |
| Osteoclast-associated immunoglobulin-like receptor | hoscar | Q8IYS5 |
| Serine protease 27 | prss27 | Q9BQR3 |
| Tissue factor | tf | P13726 |
| Receptor for advanced glycosylation end products | rage | Q15109 |
| Tumor necrosis factor receptor superfamily member 13B | tnfrsf13b | O14836 |
| Lymphotactin | xcl1 | P47992 |
| Tyrosine-protein kinase Mer | mertk | Q12866 |
| Decorin | dcn | P07585 |
| Pro-interleukin-16 | il16 | Q14005 |
| Prolargin | prelp | P51888 |
| Programmed cell death 1 ligand 2 | pdl2 | Q9BQ51 |
| Brother of CDO | boc | Q9BWV1 |
| Macrophage receptor MARCO | marco | Q9UEW3 |
| Tumor necrosis factor receptor superfamily member 11A | tnfrsf11a | Q9Y6Q6 |
| Spondin-2 | spon2 | Q9BUD6 |
| Protein AMBP | ambp | P02760 |

## **Supplementary Table 12**. PC8 Top 20 Proteins: Females

| **Protein Name** | **Protein (short)** | **Uniprot** |
| --- | --- | --- |
| Leptin | lep | P41159 |
| Lipoprotein lipase | lpl | P06858 |
| Proprotein convertase subtilisin/kexin type9 | pcsk9 | Q8NBP7 |
| Prostasin | prss8 | Q16651 |
| Low-density lipoprotein receptor | ldl_receptor | P01130 |
| Cathepsin Z | ctsz | Q9UBR2 |
| Protein AMBP | ambp | P02760 |
| E-selectin | sele | P16581 |
| Macrophage receptor MARCO | marco | Q9UEW3 |
| Spondin-2 | spon2 | Q9BUD6 |
| Retinoic acid receptor responder protein 2 | rarres2 | Q99969 |
| Protein delta homolog 1 | dlk1 | P80370 |
| Interleukin-10 receptor subunit beta | il10rb | Q08334 |
| C-C motif chemokine 22 | ccl22 | O00626 |
| Pentraxin-related protein PTX3 | ptx3 | P26022 |
| Vascular endothelial growth factor D | vegfd | O43915 |
| Secretoglobin family 3A member 2 | scgb3a2 | Q96PL1 |
| Paraoxonase | pon3 | Q15166 |
| Brain-derived neurotrophic factor | bdnf | P23560 |
| Insulin-like growth factor-binding protein 2 | igfbp2 | P18065 |

## **Supplementary Table 13**. PC1 Top 20 Proteins: Males

| **Protein Name** | **Protein (short)** | **Uniprot** |
| --- | --- | --- |
| CD166 antigen | alcam | Q13740 |
| Intercellular adhesion molecule 2 | icam2 | P13598 |
| Granulins | grn | P28799 |
| C-X-C motif chemokine 16 | cxcl16 | Q9H2A7 |
| Insulin-like growth factor-binding protein 7 | igfbp7 | Q16270 |
| Lymphotoxin-beta receptor | ltbr | P36941 |
| Tyrosine-protein phosphatase non-receptor type substrate 1 | shps1 | P78324 |
| Contactin-1 | cntn1 | Q12860 |
| Tyrosine-protein kinase receptor UFO | axl | P30530 |
| Aminopeptidase N | apn | P15144 |
| Perlecan | plc | P98160 |
| Osteoprotegerin | opg | O00300 |
| Epidermal growth factor receptor | egfr | P00533 |
| Interleukin-1 receptor type 2 | il1rt2 | P27930 |
| Interleukin-1 receptor type 1 | il1rt1 | P14778 |
| Tumor necrosis factor receptor 1 | tnfr1 | P19438 |
| Interleukin-6 receptor subunit alpha | il6ra | P08887 |
| Tartrate-resistant acid phosphatase type 5 | trap | P13686 |
| Matrix metalloproteinase-2 | mmp2 | P08253 |
| Interleukin-18-binding protein | il18bp | O95998 |

## **Supplementary Table 14**. PC7 Top 20 Proteins: Males

| **Protein Name** | **Protein (short)** | **Uniprot** |
| --- | --- | --- |
| Leptin | lep | P41159 |
| Low-density lipoprotein receptor | ldl_receptor | P01130 |
| E-selectin | sele | P16581 |
| Protein delta homolog 1 | dlk1 | P80370 |
| Tissue-type plasminogen activator | tpa | P00750 |
| Transferrin receptor protein 1 | tr | P02786 |
| Prostasin | prss8 | Q16651 |
| Plasminogen activator inhibitor 1 | pai | P05121 |
| Lipoprotein lipase | lpl | P06858 |
| Cathepsin Z | ctsz | Q9UBR2 |
| Interleukin-18 receptor 1 | il18r1 | Q13478 |
| P-selectin glycoprotein ligand 1 | psgl1 | Q14242 |
| Proprotein convertase subtilisin/kexintype9 | pcsk9 | Q8NBP7 |
| Protein AMBP | ambp | P02760 |
| Interleukin-27 | il27 | Q14213_Q8NEV9 |
| Secretoglobin family 3A member 2 | scgb3a2 | Q96PL1 |
| Growth hormone | gh | P01241 |
| Osteopontin | opn | P10451 |
| Insulin-like growth factor-binding protein 1 | igfbp1 | P08833 |
| Insulin-like growth factor-binding protein 2 | igfbp2 | P18065 |
